# First-Person Perspective Virtual Body Posture Influences Stress: A Virtual Reality Body Ownership Study

**DOI:** 10.1371/journal.pone.0148060

**Published:** 2016-02-01

**Authors:** Ilias Bergström, Konstantina Kilteni, Mel Slater

**Affiliations:** 1 Event Lab, Facultat de Psicologia, Universitat de Barcelona, Barcelona, Spain; 2 Department of Computer Science, University College London, London, United Kingdom; 3 Institició Catalana de Recerca i Estudis Avançats (ICREA), Barcelona, Spain; Birkbeck, University of London, UNITED KINGDOM

## Abstract

In immersive virtual reality (IVR) it is possible to replace a person’s real body by a life-sized virtual body that is seen from first person perspective to visually substitute their own. Multisensory feedback from the virtual to the real body (such as the correspondence of touch and also movement) can also be present. Under these conditions participants typically experience a subjective body ownership illusion (BOI) over the virtual body, even though they know that it is not their real one. In most studies and applications the posture of the real and virtual bodies are as similar as possible. Here we were interested in whether the BOI is diminished when there are gross discrepancies between the real and virtual body postures. We also explored whether a comfortable or uncomfortable virtual body posture would induce feelings and physiological responses commensurate with the posture. We carried out an experiment with 31 participants in IVR realized with a wide field-of-view head-mounted display. All participants were comfortably seated. Sixteen of them were embodied in a virtual body designed to be in a comfortable posture, and the remainder in an uncomfortable posture. The results suggest that the uncomfortable body posture led to lesser subjective BOI than the comfortable one, but that participants in the uncomfortable posture experienced greater awareness of their autonomic physiological responses. Moreover their heart rate, heart rate variability, and the number of mistakes in a cognitive task were associated with the strength of their BOI in the uncomfortable posture: greater heart rate, lower heart rate variability and more mistakes were associated with higher levels of the BOI. These findings point in a consistent direction—that the BOI over a body that is in an uncomfortable posture can lead to subjective, physiological and cognitive effects consistent with discomfort that do not occur with the BOI over a body in a comfortable posture.

## Introduction

Imagine being seated on an aeroplane in an uncomfortable posture, but through wearing a head-mounted display you see in immersive virtual reality that the life-sized virtual body that substitutes your own is in a comfortable posture. Would this induce feelings of comfort in spite of your actual uncomfortable posture? A body ownership illusion (BOI) is the perceptual illusion that artificial body parts or full bodies can be perceived by healthy adults as their own, an issue that is a current topic in the neuroscience of body representation [1–4]. In this paper we address the question of whether the posture of a life-sized virtual body that is seen by participants from first person perspective (1PP), and that is different from their actual posture, affects the level of the BOI and also whether it influences feelings of comfort and discomfort and corresponding physiological state in the participants.

A classic example of a BOI is the rubber hand illusion (RHI), where participants see a rubber hand close to their own occluded hand, and the experimenter strokes both rubber and real hands synchronously at homologous areas [5]. After a few seconds of such visuotactile stimulation, the majority of participants perceive the rubber hand as if it were their own [6]. It has also been shown that the RHI can be induced when both rubber and real hands move synchronously in time [7–9], or when they are apparently collocated [10]. Using similar methods, BOIs have also been demonstrated towards full humanoid mannequins. For example, participants see a mannequin at the same location as their own body (as if collocated), through a head mounted display (HMD), and from 1PP. When their real body is stroked in synchrony with strokes seen to be applied to the mannequin body then they have a BOI with respect to that body [11, 12].

Several experiments have shown that BOIs can be induced towards virtual body parts or bodies in IVR. For example, a virtual version of the RHI was shown to function with similar intensity as the RHI, towards a virtual arm and hand that was seen to be touched synchronously with the participants’ real hand [13]. Similarly, participants perceived a virtual hand as if it were their own when this was seen to move synchronously [14] with their real hand movements and when receiving visuomotor and tactile feedback synchronously with their real hand [15]. Furthermore, BOIs were induced also towards virtual bodies when these were seen to substitute the participants’ bodies under synchronous visuotactile, visuomotor or both types of stimulation [15–22].

BOIs in both physical and virtual reality have been shown to have physiological consequences for the participants’ real counterparts. For example, owning a fake hand decreases the temperature [23, 24] and changes the temperature sensitivity of the real hand [25] and increases the histamine reactivity in the real arm [26]. Moreover, a threat to the fake body while experiencing the illusion produces higher skin conductance responses [11] and heart rate deceleration [16] compared to when the illusion is not experienced, with motor cortex activation in response to an attack on the virtual body that would be expected in response to an attack on the real body [27].

In a case study described in [28] we found that when healthy comfortably seated participants are embodied in a virtual body seen from 1PP and in a mirror, in a posture that indicates stress (that has been reportedly used in interrogations), they tend to report feelings of bodily discomfort. In the current study we aimed to test whether virtual body posture seen from 1PP and in a mirror would influence the illusion of body ownership of participants, their feelings of comfort or discomfort, and associated physiological responses.

Thirty-one participants were recruited for the experiment, 16 were embodied in a virtual body in a comfortable posture (condition Comfort), and another 15 in a body with an uncomfortable posture (condition Discomfort), in this between-groups single factor design. There were 5 males in each group. The mean (and SD) age of the Comfort group was 21 ± 3, and the Discomfort group 24 ± 11. The experiment was approved by the Comisión de Bioética de la Universitat de Barcelona and carried out in accordance with that approval. Participants gave written informed consent.

**Fig 1:**
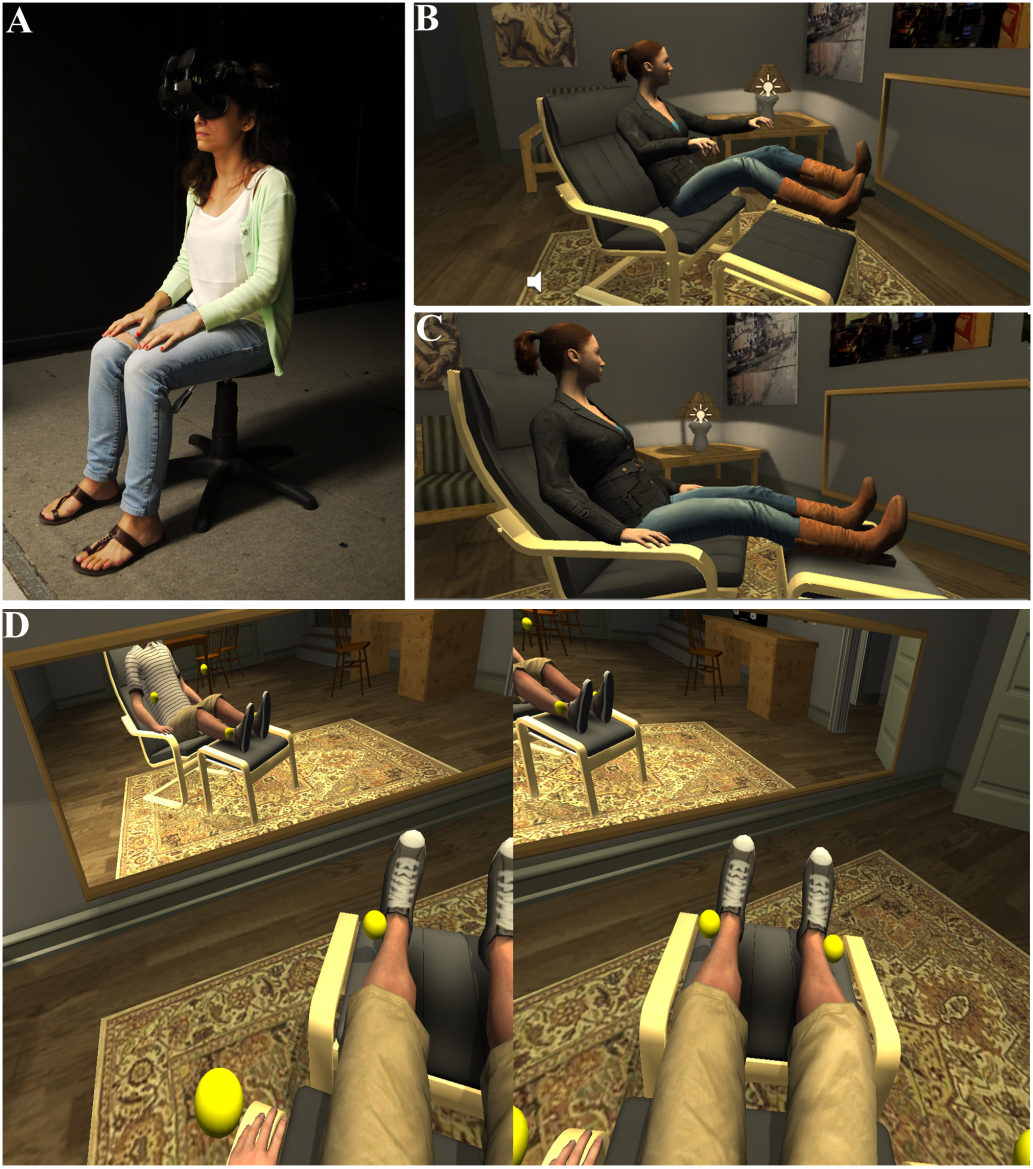
The experimental scenario. (A) The actual posture of the participant. (B) The posture in the Discomfort position seen from a third person perspective (C) The posture in the Comfort condition seen from third person perspective (D) First person perspective view of the male virtual body in the Comfort condition. Note that the head was never visible in the mirror.

Virtual embodiment was achieved through a head-tracked wide field-of-view stereo head-mounted display (HMD). All participants first experienced a baseline period for 5 minutes where they were not represented by a virtual body. Then they saw their virtual body from 1PP in either the comfortable or uncomfortable posture (Fig 1). In order to enhance the likelihood that they would experience the BOI virtual balls were programmed to touch the virtual hands and feet of the virtual body, collocated with the participant’s real limbs, synchronous with short firings of vibrotactile devices at the corresponding position on the real body. Hence embodiment was achieved through both 1PP and through synchronous visuo-vibrotactile stimulation for 5 minutes. See Methods for a full description of the setup and procedures, and S1 Video.

## Results

### Response variables

Subjective (questionnaire) responses on BOI, comfort and discomfort were elicited, and various behavioural and physiological data were recorded. In order to record feelings of comfort and discomfort frontal and dorsal body maps were displayed in the HMD, for participants to report their comfort and discomfort per body part. These were displayed immediately after the baseline and main experiment condition. The body map used was adapted from [29]. A 2D image of a body appeared in front of participants. As each of the 20 body parts blinked in turn, participants were asked to say out loud the level of discomfort experienced during the past five minutes for that body part, on a scale from 1 (no discomfort “I experience no unpleasant sensations” through 4 (moderate discomfort, “affects my ability to concentrate”), to 7 (agony “impossible for me to endure”). The above cycle was then repeated, for all body parts, gauging instead the level of comfort experienced, on a scale from 1 to 7, where 1 (no comfort, “I am experiencing great agony”), through 4 (moderate, “While comfortable, I experience distracting sensations I’d rather be without”) to 7 (“The greatest level of comfort possible”). TotalDiscomfortBase and TotalComfortBase refer to the sums of all 20 responses for the baseline, and TotalDiscomfort and TotalComfort refer to the sums for the experimental period. However, instead of using these raw scores we make use of Item Response Theory (IRT) to produce latent scores that are adjusted to take account of the differing relevance of the 20 items, and also the potential differing individual susceptibility. We call these latent scores LDiscomfortBase, LDiscomfort, LComfortBase, and LComfort, respectively, where the L stands for ‘latent’. These latent scores each range on a continuous scale between -4 and +4 as standard normal variables. See Methods for further details. Here we concentrate on the comfort scores, and the discomfort scores are considered in Section F of S1 File.

Participants carried out a cognitive task during the baseline and the experimental manipulation that required them to count backward by threes for 60 seconds starting from a pseudo-randomly chosen three-digit number. Previous research has shown this task to be an effective elicitation of attentional demand [30, 31]. Performance on the cognitive task was measured by the total number of correct responses (CountBase, Count).

Electrodermal activity and ECG were recorded throughout the experiment. However, the electrodermal data was unreliable due to faulty recordings on several participants, and therefore are not further discussed. ECG was measured as the mean heart rate and heart rate variability during the middle 4 minutes (thus removing first and last 30 seconds) in the baseline and experimental manipulations. HRBase and HR are the mean instantaneous heart rate in the baseline and experimental conditions. Heart rate variability is measured by the NN50, which is the number of pairs of successive normal-to-normal heart beats that differ by more than 50 ms [32], referred to as NN50Base for the baseline and NN50 for the manipulation period. Increased HR and decreased HRV are signs of illness or stress (e.g. [33], and [34] for an application in virtual reality).

Subjective physiological response was assessed by the Autonomic Perception Questionnaire (APQ), which is a 24 item visual analogue scale used for the assessment of self-awareness of physiological activation (heart rate, perspiration, temperature change, respiration, gastro intestinal, muscle tension and blood pressure). A high score on the APQ indicates greater awareness of bodily sensations and correlates positively with anxiety, heart rate and skin conductance response [35]. Participants completed this questionnaire before the VR experience (APQPre) and after the VR experience (APQ). One missing response was replaced by the mean of all other responses in the same experimental condition.

The illusion of body ownership was assessed by several questions (Table A of S1 File), the most important being MeDown (“Although the virtual body that I saw did not look like me, I felt as if the body I saw when looking down might be my body”) and MeMirror (“Although the virtual body that I saw did not look like me, I felt as if the body I saw when looking in the mirror might be my body”). These were scored on a 1–7 Likert scale with 1 indicating no agreement and 7 maximum agreement to the corresponding statement. MeDown is considered here, and MeMirror in Section F of S1 File. One control question was Another (“In general I felt that the body belonged to someone else.”). Several other questions are described in Table A of S1 File.

### Statistical Model

The formal (Bayesian) statistical model used is described in Section B of S1 File. We treat only the critical variables—subjective body ownership and comfort responses, the APQ, heart rate, NN50, and the number of correct responses in the counting task. It should be noted that this is one overall model, where all stochastic equations are treated simultaneously rather than as a series of separate analyses. In other words the Bayesian method returns the joint posterior distribution of all the model parameters. Note that all prior distributions on the model parameters were chosen to be non-informative, that is, with very large variance (Section B of S1 File) and heavily biased against our hypotheses. Analysis was carried out using the JAGS system [36], together with MATLAB using MATJAGS (http://psiexp.ss.uci.edu/research/programs_data/jags/), and some graphs were produced using Stata 14.

### Body ownership illusion

In this experiment participants were never in the same posture as their virtual body even though they saw the virtual body from first person perspective with respect to the virtual body’s eyes (Fig 1). The participant was always seated, but the virtual body was reclining in both Comfort and Discomfort conditions (Fig 1B and 1C). See Methods (Experimental Design) for further discussion of this point. Here we consider the extent of subjective body ownership and whether it differed between the Comfort and Discomfort conditions.

**Fig 2:**
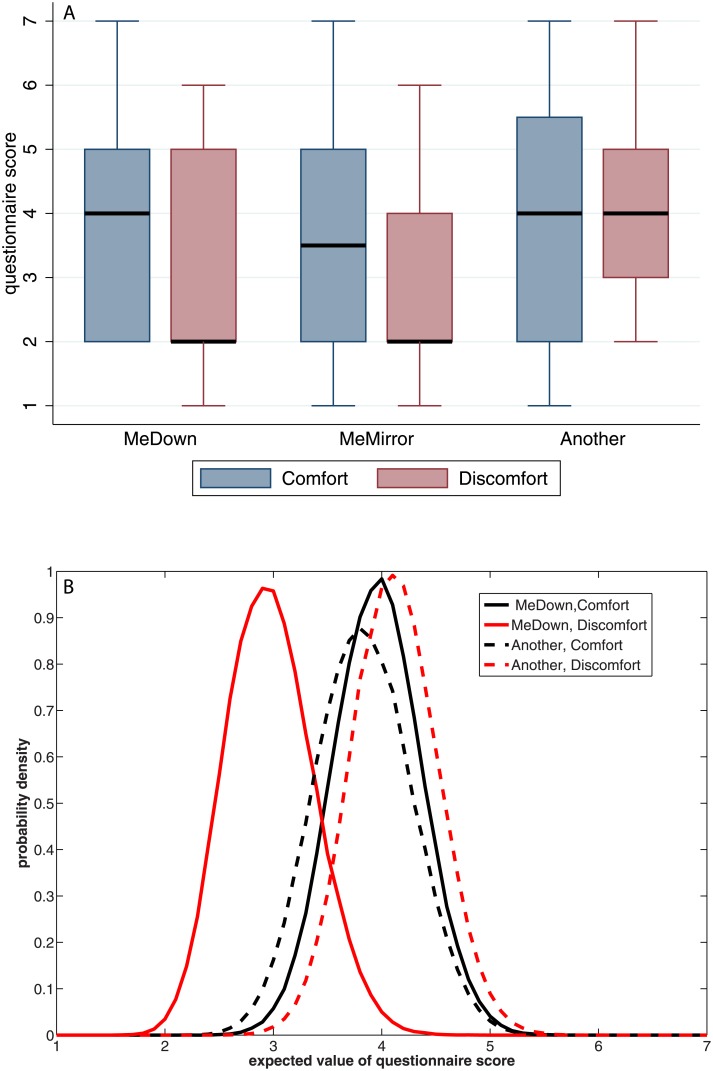
Body ownership illusion questions MeDown and Another by Condition. (A) Box plots (B) Posterior distributions of the expected values of the BOI questionnaire scores. This is based on the ordinal logistic regression.

Fig 2A shows the boxplot of the BOI questionnaire scores by the body posture condition. Compared to results from other studies—e.g. [20]—the median scores on MeDown and MeMirror are generally not high and do not seem different to the non-ownership question scores (Another). Additionally, the Comfort condition results in higher BOI than the Discomfort condition.

The overall statistical model includes a logistic regression of the ordinal questionnaire scores (MeDown, Another) on the experimental condition (where we code Comfort = 0 and Discomfort = 1). From this we can obtain the posterior distributions of the expected values (means) of MeDown and Another, which are shown in Fig 2B. This supports the finding that the illusion of ownership is less in the Discomfort condition than in the Comfort. For example, for MeDown the posterior probability that the mean of MeDown > 4 is 0.46 in the Comfort condition and 0.009 in the Discomfort condition. The equivalent probabilities for Another are 0.35 and 0.61. In the Discomfort condition the probability is far greater for higher scores of Another than MeDown, as can also be observed from Fig 2B.

### Comfort and Discomfort postures and subjective and physiological responses

Our hypothesis with respect to the influence of the virtual body posture is that the Discomfort condition compared to Comfort would result (relative to baseline) in: lower feelings of comfort, greater APQ, greater heart rate and lower heart rate variability, and less success in the counting backwards task. Table 1 shows the results of the statistical analysis with respect to each of these, and we now consider each in turn.

**Table 1:** Results of the Statistical Analysis on all the responses related to Comfort. Throughout *B* refers to Baseline, e.g. *C* denotes LComfort with respect to the experimental period and *CB* denotes the Baseline variable LComfortBase. X is the experimental condition (X = 0 Comfort, X = 1 Discomfort).

Response Variable, individual *i*	Link between mean and linear model	Hypothesis (H) on the parameter of interest	Posterior Distribution of parameter of interest	P(H|D).D = data.Priors = 0.0013	Interpretation
**MeDown** *O*_*i*_	*μ*_*Oi*_ = *β*_*O*0_ + *β*_*O1*_X_*i*_	*β*_*o1*_ < 0	Figure A in S1 File	0.95	Strong evidence that Ownership is less in the Discomfort condition.
**Another** *NO*_*i*_	*μ*_*NOi*_ = *β*_*NO*0_ + *β*_*NO*1_X_*i*_	*β*_*No*1_ > 0	Figure B in S1 File	0.69	Some evidence that Non-ownership may increase in the Discomfort condition.
**LComfort** *C*_*i*_	*μ*_*Ci*_ = *CB*_*i*_ + *β*_*C*0_ + *β*_*C*1_X_*i*_ $C_{i}∼N(\mu_{i},\sigma_{C}^{2})$	*β*_*C*1_ < 0	Figure C in S1 File	0.93	Strong evidence that Comfort is less relative to the baseline in the Discomfort condition.
**APQ** *A*_*i*_	*μ*_*Ai*_ = log(*AB*_*i*_) + *β*_*A*0_ + *β*_*A*1_X_*i*_ $\text{log}(A_{i})∼N(\mu_{Ai},\sigma_{A}^{2})$	*β*_*A*1_ > 0	Figure D in S1 File	0.83	Good evidence that APQ is greater relative to baseline in the Discomfort condition.
**Heart Rate** *H*_*i*_	*μ*_*Hi*_ = *HB*_*i*_ + *β*_*H*0_ + *β*_*H*1_X_*i*_ + *β*_*H*2_ *O*_*i*_ + *β*_*H*3_X_*i*_ *O*_*i*_ $\text{log}(H_{i})∼N(\mu_{Hi},\sigma_{H}^{2})$	*β*_*H*3_ > 0	Figure E in S1 File	0.90	Strong evidence that HR is positively associated with BOI in the Discomfort condition.
**NN50** *N*_*i*_	log(*μ*_*Ni*_) = log(*NB*_*i*_ + 1)+*β*_*N*0_ + *β*_*N*1_X_*i*_*β*_*N*2_*O*_*i*_ + *β*_*N*3_X_*i*_ *O*_*i*_ $\text{log}(N_{i}+1)∼N(\mu_{Ni},\sigma_{N}^{2})$	*β*_*N*3_ < 0	Figure F in S1 File	0.99	Overwhelming evidence that NN50 is negatively associated with BOI in the Discomfort condition.
**Count** *T*_*i*_	*μ*_*Ti*_ = log(*TB*_*i*_) + *β*_*T*0_ + *β*_*T*1_X_*i*_*β*_*T*2_*O*_*i*_ + *β*_*T*3_X_*i*_ *O*_*i*_ $\text{log}(T_{i})∼N(\mu_{Ti},\sigma_{T}^{2})$	*β*_*T*3_ < 0	Figure G in S1 File	0.83	Good evidence that the number of correct counts is negatively associated with BOI in the Discomfort condition.

**Fig 3:**
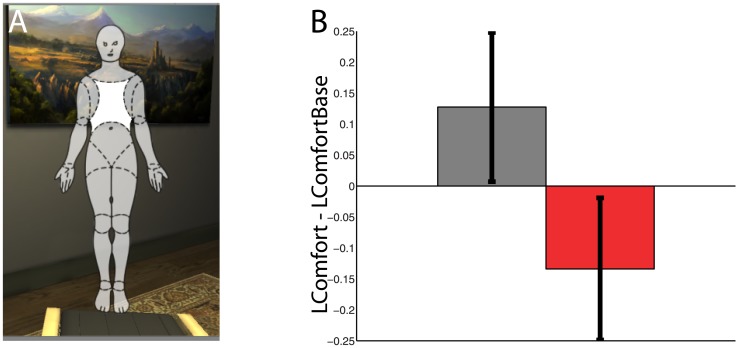
Assessment of Comfort (A) Frontal view of the body map used where each area of the body highlighted in turn and participants were asked to score their level of comfort for that body part on a 1–7 Likert scale. (B) Bar charts showing the means and standard errors of the Comfort—ComfortBaseline by Condition.

Fig 3A shows the body map that was used in the assessment of Comfort. Fig 3B shows the means and standard errors of the subjective LComfort—LComfortBase scores, indicating that the feeling of comfort decreased in the Discomfort compared to the Comfort condition. From Table 1 we can see that the posterior probability that the Discomfort condition decreases the feeling of comfort is 0.93. We also examined whether there is an interaction effect between Condition and MeDown (to check whether greater levels of body ownership were associated with a further decrease of subjective comfort in the Discomfort condition). However, the posterior distribution of the interaction term coefficient was almost symmetric about 0.

**Fig 4:**
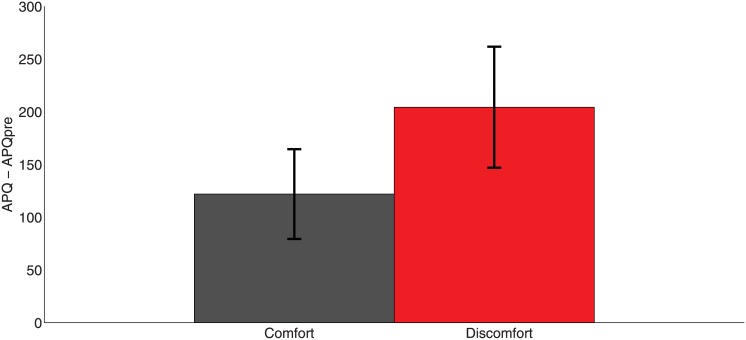
Bar chart of means and standard errors of APQ—APQpre by Condition.

Fig 4 shows that the mean level of subjective awareness of physiological responses is greater in the Discomfort compared to the Comfort condition. A scatter plot of APQ by APQpre shows heteroscedasticity which is removed by working on a log scale. From Table 1 we can see that the coefficient of Condition is positive with posterior probability 0.83. As above there is no advantage in fitting an interaction term between Condition and MeDown.

**Fig 5:**
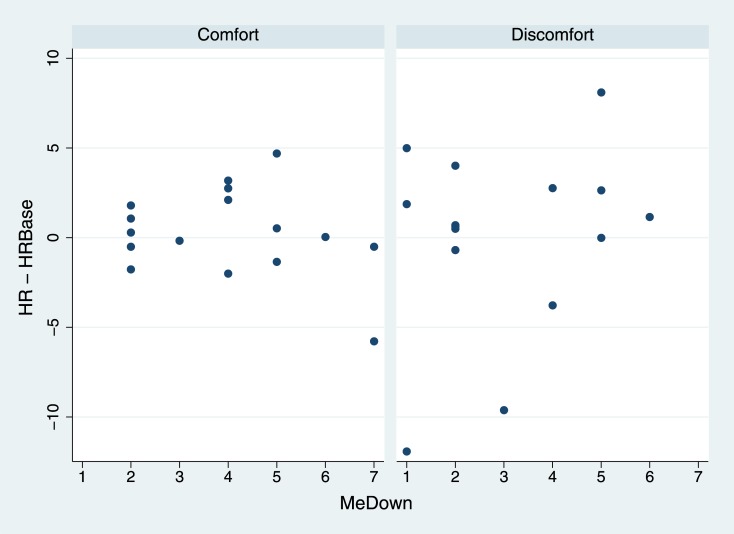
Change in Heart Rate by MeDown and Condition.

Fig 5 shows the scatter plot of HR-HRBase by the BOI question MeDown and Condition. This suggests that at least there is a different pattern of responses in the two conditions, and possibly in the Discomfort condition there is a greater change in HR the greater the BOI. Table 1 therefore includes an interaction term between MeDown and Condition. The posterior probability of the coefficient of the interaction term being positive is 0.90 suggesting that HR is positively associated MeDown in the Discomfort condition.

**Fig 6:**
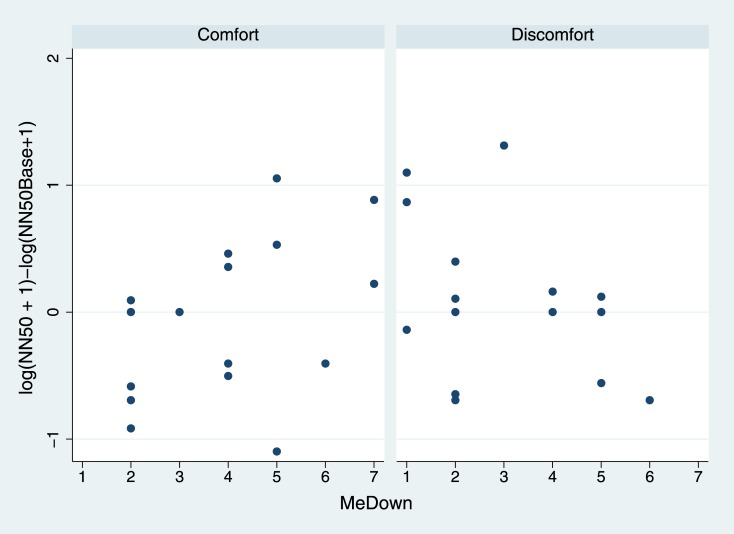
Change in NN50 (Heart Rate Variability) by MeDown and Condition. Note that the NN50 scores have been incremented by 1 for this graph in order to avoid log(0).

Fig 6 suggests that HRV as measured by NN50 is negatively associated with MeDown in the Discomfort condition. We consider the coefficient of the interaction term between Condition and MeDown. The vast amount of the posterior distribution is in the negative region.

**Fig 7:**
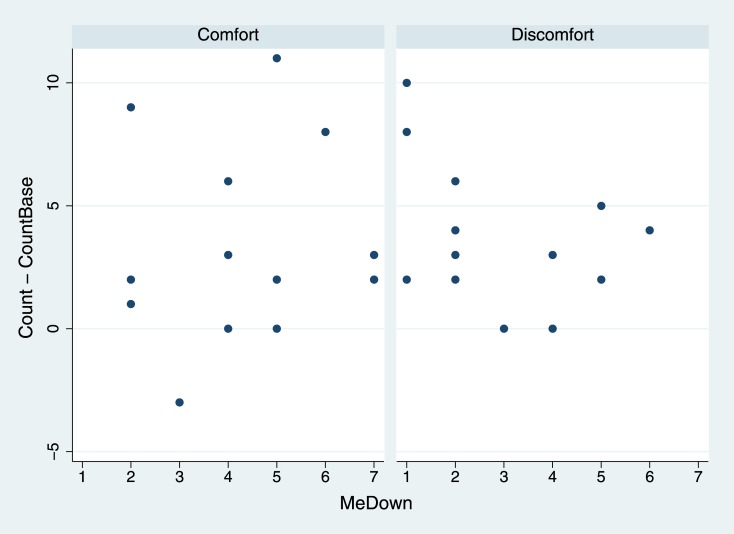
Change in counting success by MeDown and Condition.

Fig 7 suggests that there is a different pattern of responses between the Comfort and Discomfort condition in the counting task, and that in the Comfort condition greater BOI was associated with greater counting success, and the opposite in the Discomfort condition. The parameter of interest is the coefficient of the interaction term, which has posterior probability 0.83 of being negative in the Discomfort condition.

The main question of our study is whether the type of posture (comfortable or uncomfortable) would impact participant responses in relation to their bodily well being. We have presented evidence suggesting that in the Discomfort condition the subjective level of comfort is less than in the Comfort condition, the APQ is higher meaning greater awareness of autonomic physiological responses, the heart rate is higher, the heart rate variability is lower, and that participants are prone to make more errors in a counting task. Higher HR together with lower HRV suggests physiological stress.

Our statistical method allows us to compute an overall probability of this—since the Bayesian method returns the joint distribution of all the parameters. In fact we find that:

$$P\left(\left(\beta_{C1}<0)\wedge (\beta_{A1}>0)\wedge (\beta_{H3}>0)\wedge (\beta_{N3}<0)\wedge (\beta_{T3}<0\right)\right)=0.57\text{K}$$ (1)

This probability reduces considerably if we change any one of the inequality signs, as shown in Table 2. Our interpretation is that starting from a prior probability close to zero, we move to a posterior probability of the hypothesis of near 60%. However, if we restrict attention solely to the physiological responses (increased heart rate and decreased heart rate variability) then the probability is 0.89. In other words there is strong evidence that physiological response is influenced—with the Discomfort posture leading to greater stress.

**Table 2:** The Effects of Changing each one of the inequalities in Eq 1.

Change in Eq 1	Resulting Probability
*β*_*C*1_ > 0	0.04
*β*_*A*1_ < 0	0.12
*β*_*H*3_ < 0	0.06
*β*_*N*3_ > 0	0.01
*β*_*T*3_ > 0	0.12

Section C of S1 File shows the posterior distributions of the coefficients, and of the standard deviations of the model, Section D of S1 File discusses convergence, Section E of S1 File the model fits to the data, and Section F of S1 File some alternative models.

### The Structure of Comfort

**Fig 8:**
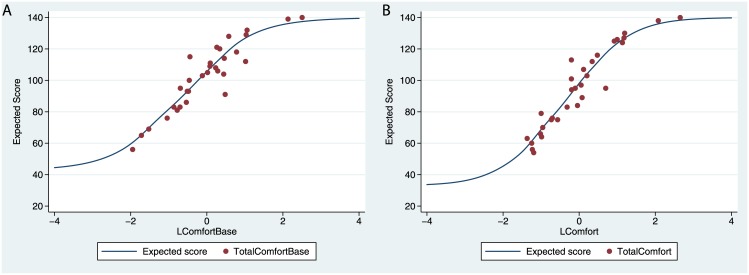
Expected comfort scores from the IRT model (A) for the Baseline and (B) for the Comfort condition.

**Fig 9:**
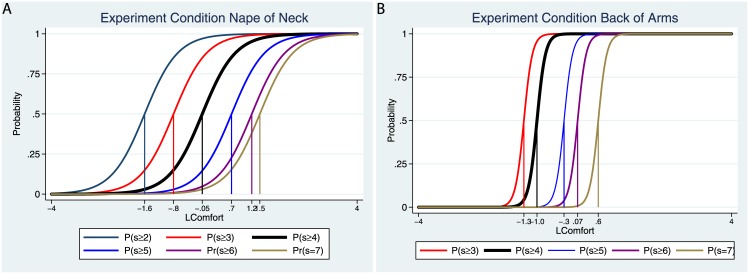
Boundary characteristic curves for (A) the nape of the neck and (B) the backs of the arms for the body map item questionnaire on comfort.

Fig 8 shows the relationships between the latent responses as estimated from the IRT model and the actual responses summed over all 20 comfort items in the body map, for the baseline and experimental periods (see Figure R in S1 File for the equivalent graphs for the discomfort scores). It can be seen that the model fits the data very well. The particular advantage of using IRT for the measurement of latent comfort is that it enables us to examine the relative contributions of each of the 20 items. This is achieved through the ‘discrimination’ parameter of the IRT model, which represents the rate of change in the probability of a higher score as a function of the underlying latent variable around the ‘break even’ probability of 0.5. Fig 9 shows the boundary characteristic curves for two different items on the body map, the nape of the neck and the backs of the arms. These curves show how the probability of the scores (s) on the items vary with the underlying latent score.

The vertical lines show estimates of the ‘difficulty’ parameters. For example, to obtain a score of at least 6 with probability 0.5 for the nape of the neck requires a latent comfort of 1.2, whereas for the backs of the arms only 0.07. Hence it is more difficult to obtain a higher score for the nape of the neck than it is for the backs of the arms. However, a small changes in latent score leads to a much greater change in probability in the case of the backs of the arms than in the case of the neck. This is the ‘discrimination’ parameter, the slopes of the curves at the probability 0.5. Consider, for example, the curves for P(s ≥ 4) (the probability at the mid-point of the item scales and above). In the case of the nape of the neck a small change in latent comfort is associated with also a small change in probability. However, in the case of the backs of the arms, a small change in latent comfort is associated with a large change in probability. Here if the underlying level of comfort drops slightly the probability of a higher score reduces dramatically.

Table 3 shows the estimates of the discrimination parameter for each of the 20 items in the baseline and experimental conditions. These show that this latent measure takes into account that the sense of comfort does not attribute equal weighting to the various items.

**Table 3:** Estimates and their Standard Errors of the Discrimination Parameter for each of the 20 Items in the Body Map for the Comfort Questions. D(s≥6) is the rank order of the difficulty parameter for obtaining a score of at least 6 from the IRT model, where 1 is the most and 20 the least difficult.

Baseline				Experiment			
	Coef	S.E.	D(s≥6)		Coef	S.E.	D(s≥6)
Backs of arms	7.79	4.43	17	Backs of arms	9.24	4.68	13
Arms	6.52	2.89	9	Backs of legs	8.39	3.73	19
Backs of legs	4.01	1.22	19	Legs	6.19	2.04	20
Legs	2.93	0.88	15	Arms	5.21	1.58	16
Backs of hands	2.91	0.96	5	Backs of thighs	2.62	0.75	8
Backs of thighs	2.53	0.77	16	Thighs	2.34	0.69	11
Chest	2.34	0.74	11	Nape of neck	2.30	0.65	1
Hands	2.29	0.75	7	Backs of feet	2.22	0.67	15
Backs of feet	2.22	0.74	10	Feet	2.19	0.69	18
Upper back	1.76	0.59	3	Abdomen	2.17	0.66	14
Backs of shoulders	1.71	0.55	6	Backs of hands	2.16	0.66	17
Abdomen	1.70	0.59	20	Shoulders	2.12	0.61	6
Lower back	1.70	0.56	4	Hands	2.07	0.63	9
Feet	1.65	0.59	14	Back of shoulders	2.00	0.60	7
Buttocks	1.65	0.56	13	Buttocks	1.82	0.57	12
Thighs	1.61	0.57	18	Chest	1.77	0.57	10
Shoulders	1.54	0.56	12	Upper back	1.74	0.52	2
Head	1.18	0.42	1	Lower back	1.71	0.54	5
Nape of Neck	1.11	0.44	2	Neck	1.70	0.52	4
Neck	0.80	0.39	8	Head	1.29	0.44	3

Another way to consider this is that although it is quite easy to get a high score for comfort for the backs of the arms, if the situation reaches a point where even the backs of the arms are not comfortable then the score can drop very sharply. On the other hand it is more difficult to get a high score for the head to be comfortable, but small changes do not change the score much. The relationship between difficulty and discriminability can be seen by comparing the D columns of Table 3 with the discrimination estimations. See Table D in S1 File for the discomfort analysis.

## Discussion

Our study was concerned with two questions. The first was whether the posture of the embodied virtual body would influence the level of subjective body ownership. The answer appears to be that it does, and that in particular the uncomfortable posture reduces the level of the illusion as represented by the questionnaire scores (see also Section F of S1 File, for analysis of MeMirror). However, it is also the case that even within the Discomfort condition 27% of the scores were 5 or more out of 7, so that some individuals may achieve a BOI even in this case. Moreover the equivalent number in the Comfort condition is 37%. Recalling that in both conditions the true posture was different from the observed posture seen from 1PP this suggests that it is possible for some participants to maintain a body ownership illusion in these conditions.

However, the overall scores of body ownership in both conditions were relatively low, especially when compared to previous studies on BOIs using IVR [19–22] including conditions where the virtual body was static, and received synchronous visuotactile stimulation [17]. Although in our study the virtual body was seen from a 1PP, its posture in both conditions was different from that of participants. For example, while the participants’ hands were resting on their knees, the virtual ones were seen either as resting comfortably on the sides of the chair or extending straight ahead without any support. The same was also true for the legs; participants were sitting with their feet on the floor while the virtual ones were seen as either resting on a footrest, or raised in the air.

Small discrepancies in position and orientation have been shown to not affect BOIs. For example, evidence from the RHI studies suggests that when the rubber hand is placed in an anatomically plausible posture but in a different position [37, 38] or orientation [39–41] from the real one, synchronous visuotactile stimulation can overcome the spatial mismatches and induce the illusion. Similarly, it is possible to induce a BOI towards a virtual body seen from a laterally shifted visual perspective with partial collocation with the real body [42], or towards a mannequin or virtual body seen from a 1PP but tilted away from the body [43, 44]. Nevertheless, when introducing discrepancies in both position and orientation, illusory tactile sensations towards the rubber hand were reported to gradually decrease in intensity with effects also in illusion onsets [45]. Therefore, our present results extend those of Lloyd from the rubber hand to a full body; high spatial discrepancies between the seen and felt postures of individual body parts can on the average attenuate the illusion.

The analysis leading to Table 2 suggests that the experiment does lend some support to the earlier case study which had pointed to the possibility that seeing a body from 1PP in a posture more uncomfortable than that of the real one, does have a negative physiological impact on comfort [28]. For practical and ethical reasons, it is not possible to ask people to stay in an actual uncomfortable posture for the 20 minutes that the experiment takes, and thus we instead chose the strategy of asking participants to sit in a neutral position, with variations in the virtual position of comfortable or uncomfortable. We cannot therefore conclude from this experiment that had participants actually been sitting in an uncomfortable posture, seeing the virtual body in a more comfortable one would have reduced their level of discomfort.

We found decreased heart rate variability (HRV) as measured by NN50 associated with higher levels of subjective body ownership in the Discomfort condition. Decreased HRV is a strong marker for increased stress, which is our most well supported result in terms of posterior probability. For a general review of HRV methods and applications see [46]; its relationship with perceived stress is shown in [33], with discomfort caused by different levels of thermal comfort in [47], and discomfort caused by the pressure of clothing in [48]. To our knowledge there are no specific studies concerned with comfort with respect to the types of postures we have used. However, it is known that participants in a supine posture tend to have greater HRV than in a standing posture as shown in Table 1 of [49], and see also [32]. Moreover lower HRV is known to be associated with greater mental workload [50], for example NN50 in particular has been shown to be particularly adept in distinguishing between different levels of mental workload in a driving task [51]. This is consistent with our finding that overall the evidence does suggest that higher body ownership in the uncomfortable posture led to physiological changes compatible with what would be expected from the literature on HRV.

The body map measure relying on 20 items each scored on a 1 to 7 scale as to their level of comfort (or discomfort) does not take into account the different contributions of items to the overall feeling of comfort. In particular, in the experiment participants wore a heavy head-mounted display that would have led to real discomfort for a number of items—especially the head and neck—irrespective of the experimental condition. Therefore we turned to IRT as a way to normalize across these items, and allow for the differing latent susceptibilities to feelings of comfort across the individuals. The analysis leading to Table 3 indeed shows the different impact of the various items.

It can be argued that a problem with our findings is that our experimental design does not permit a conclusion that the impact of the differing virtual body postures on the level of subjective comfort and associated physiological responses are actually due to the body ownership illusion. To be safer we would have needed an experimental condition that was specifically designed to elicit low subjective body ownership—such as a third person perspective, or an asynchronous visuotactile condition. However, in fact we had sufficient variation in the subjective scores on body ownership as to allow the analysis of its impact on the various responses, and Table 2 shows that only in the Discomfort condition is it positively associated with Heart Rate, negatively with NN50 and negatively with Count. Moreover Table C in S1 File shows that the relationship between the physiological and count scores does not hold when the control question (Another) is used in place of the BOI questions (MeDown, MeMirror). Therefore the evidence does suggest that it specifically the BOI that is behind these findings.

It can furthermore be argued that we cannot fully control for any possible effects of the similarity or difference between the actual posture of the participant and the comfortable and uncomfortable virtual postures. In other words it is impossible to ensure that the real posture is neutral in comfort between the two virtual postures. However, our design has attempted to minimize such an effect, by maintaining a similar body topology for both, and by ensuring that all limbs can be looked at by the participant with a similar degree of ease between the two postures. The two postures obviously vary, however, in the amount of effort needed to maintain them.

Regarding the postures is important to note that while the actual seating posture may appear quite comfortable, there were several factors which in fact contributed towards participant discomfort: participants wore a heavy head mounted display; they could not move to relieve fatigue, during each of the baseline/experiment conditions; they did not rest their back anywhere in the real posture; and, finally, while their palms rested on their thighs, this did not relieve the weight of the arms from having to be supported by the back. Thus although it might be thought that the Comfortable condition posture was quite close to the actual posture, this was not the case. There is evidence for this in the question (MyPosture) “There were moments in which I felt as if my body was in the same posture as that of the virtual body”. As shown in Table A in S1 File the distributions of scores are almost the same, and overall are low, for the Comfort and Discomfort conditions, which would not be the case were one of the postures closer to the true posture of the participants.

In conclusion our results suggest that manipulating the body posture of people’s virtual body representations to be different to their actual posture, can lead to a lesser perception of body ownership compared to results of studies where the real and virtual postures are matched. Nevertheless a posture more uncomfortable than the real one can result in a psychologically and physiologically detectable experience of discomfort.

## Methods

### Participants

Thirty-one participants were recruited through advertising around the campus. Another two had been recruited but were excluded due to technical failures. None of the participants had any prior knowledge of the experiment. The study was performed according to institutional and national ethical standards for the protection of human participants. All participants were compensated with 10 euros after the end of the experiment. The final distribution of participants is shown in Table 4. No significant differences between groups were found in age or other demographic data gathered.

**Table 4:** Distribution of Participants by Condition, Mean and S.E. of Age.

	Gender		
Body Posture	Male	Female	Total
Comfort			
Mean age	21	20	
S.E.	1.4	1.1	
	n = 5	n = 11	n = 16
**Discomfort**			
Mean age	23	25	
S.E.	1.7	4.2	
	n = 5	n = 10	n = 15

The study was approved by and carried out in accordance with the regulations of the Comisión de Bioética de la Universitat de Barcelona, and was therefore performed in accordance with the ethical standards laid down in the 1964 Declaration of Helsinki. Participants gave written informed consent on a form devised for this purpose that had been approved by the said Comisión de Bioética. The individual shown in Fig 1 in this manuscript has given written informed consent (as given in the PLOS consent form) to publish this Figure.

### Experimental design

The experimental design was between groups with one factor Condition, which was the virtual body posture with the two levels Comfort and Discomfort. At the start of the experiment, participants experienced a Baseline condition, where they were not represented by a virtual body. Then depending on their assigned group, participants experienced either the Comfort or Discomfort condition.

Considerable effort was expended in choosing the three seating postures to be used—i.e. the actual (Fig 1A), the virtual uncomfortable (Fig 1B), and virtual comfortable (Fig 1C) postures. All three were varied in pilot experiments, with the aim of selecting three for which the actual posture would be experienced as being neutral with respect to the two virtual postures in experience of comfort, and in perceived topological similarity—i.e., in particular less comfortable than the Comfort condition and more comfortable than the Discomfort condition. It was also important that in both virtual postures, the virtual arms and legs would be positioned similarly in relation to the first-person perspective view, so that they would appear in approximately the same areas within the participant’s view in both uncomfortable and comfortable virtual postures, and therefore could be looked at with a similar degree of ease.

To ensure that the postures used were physically possible, members of our team who exercise regularly were recruited to try and maintain the uncomfortable posture; this was indeed possible for a few minutes, albeit of course with considerable effort.

### Materials

The virtual scenario was implemented using the Unity3D software; the virtual bodies were animated using the Motion Builder character animation software, to show the slight variation in motion a person would exhibit when sitting still in each of the poses, while 3D models were created using the 3D Studio MAX software.

The head-mounted-display (HMD) used was the NVIS nVision SX111. This displays a 3D scene in stereo with a horizontal field of view of 102 degrees and vertical field of view of 64 degrees by sending left-eye and right-eye images to left and right hand display screens. Its weight is 1.3Kg. A six degree of freedom (6DoF) Intersense IS900 motion tracker is mounted on top, the data from which continuously updates the orientation of the participant’s viewpoint, creating the sensation that they were using their head gaze normally to look around the virtual scene. For audio reproduction we used a Yamaha Digital Sound Projector YSP-4000 powered loudspeaker, participants being seated in such a way that the speaker was collocated with the virtual reality scenario window through which environment sound emanated.

Four feedback devices were each attached on the back of the palms and the bottom of shins respectively (see Fig 1D), to provide vibrotactile stimulation, so as to enhance the experience of virtual embodiment. The vibration of these devices was triggered by the touching of the virtual avatars of four randomly moving yellow balls, collocated on the virtual avatar with the vibrators on the participant’s real body.

The haptic interface used was repurposed from a device developed internally in our lab [52]. The configuration used comprises of an array of four vibrators and an Arduino MEGA microcontroller board. The vibrators were coin type vibrators, encapsulated within a metal casing so that no moving parts come in contact with the user’s body. The vibrators were mounted on small boards that can be attached using adhesive Velcro strips directly to the skin.

The electrophysiological measurement data was captured using the g.USBAmp device (g.tec, Guger Technologies OEG, Graz, Austria). Bipolar ECG was measured by placing three electrodes on the left and right collarbones and the lowest left rib of each participant. Furthermore, a piezo-crystal respiration effort sensor from SleepSense was placed on the upper part of the chest to record respiratory measures. Finally, electrodermal activity (EDA) was measured by placing two electrodes in the palmar areas of the index and ring fingers of the right hand. The g.USBAmp was integrated into a real-time system using Simulink (Simulink, 2012) to store physiological data at a sample rate of 256 Hz. Offline analysis of the physiological signals was carried out using the gBSanalyze program from g.tec, as well as using custom MATLAB (Mathworks, Inc., Natick, MA) scripts.

### Procedures

When participants arrived, they were randomly assigned to a condition using an order created using the online research randomizer tool (http://www.randomizer.org/). They were given an information sheet to read, after which experimental procedures were also explained to them verbally, to ensure that they have understood it. They read and signed an informed consent form. They were given the APQ questionnaire pre-test to fill in. Participants were in all conditions asked to sit on a stool with no back support, to provide for a seating position that is approximately between the two extreme postures of the virtual body posture for Comfort and Discomfort conditions (Fig 1A).

They were assisted to don the HMD, calibrated so that its two screens were symmetrically placed over the participants’ eyes using the method described in [53], the electrophysiological measurement equipment was attached, they were assisted to sit in the required posture on the stool, and finally the vibrotactile devices were attached to the back of the hands and the ankles. While participants maintained their eyes closed, the stool was rotated so that the direction of their real body matched that of the virtual body in the simulation. The VR area of the laboratory was closed off from the rest of the laboratory by a black curtain, so that the participants were in darkness once the experiment started.

Upon starting, participants were left to accustom themselves to the displayed environment for 1 minute. During this time they were tasked with looking around them and to describe what they saw.

Each participant experienced two phases, each lasting 5 minutes, in each of which they were situated in the virtual environment. The first was always the baseline, followed by the experimental condition. During each, they were asked to pay attention to their experienced levels of physical comfort and discomfort, at each body part in turn, as they heard its name read out. They were instructed to direct their gaze directly at the body part named if possible otherwise to simply pay attention to their sensations from the body part in question. Half the time they were instructed to look at it directly, and half in the virtual mirror in front of them.

Immediately following the baseline, participants were shown the frontal and then dorsal views of a body map (Fig 3A) and were asked to report their subjective levels of comfort for each body part in turn, followed by a global rating, and then the whole procedure again, but for discomfort. After this, they carried out the cognitive task.

After a brief rest, participants experienced the experimental condition, again lasting 5 minutes, during which they either experienced the virtual comfort or virtual discomfort posture, depending on their group assignment. Subsequently, they again had to give body map ratings, and carried out the counting backwards cognitive task, in a manner identical to that right after the baseline condition.

After the experiment, they were asked to fill out three questionnaires, first one on the experience (Likert scale as well as open-ended questions), then the APQ post-test questionnaire, and finally a questionnaire to record demographic information. After this, they were paid, debriefed, and thanked for their participation.

A week after the experiment, we contacted them on email, and asked them a few follow up questions, on their impressions, and on any potential lasting effects of the experience.

### IRT Method

Item Response Theory provides a methodology for the estimation of an assumed latent variable underlying a set of responses such as in a questionnaire. A recent survey regarding its utility in clinical assessment is given in [54]. In this research we have used the Stata 14 function ‘irt grm’ which is a graded response model, designed for ordinal responses. In this model shown in Eq 2 *a*_*i*_ is the ‘discrimination’ for item *i*, *b*_*ik*_ is the ‘difficulty’ in responding to item *i* with a score of at least k, and *θ*_*j*_ is the latent value for person *j*.

$$\begin{array}{l} P(Y_{ij}\geq k)=\frac{1}{1+e^{-a_{i}(\theta_{j}-b_{ik})}},\theta_{j}:N(0,1)\text{K}\\ i=1,\ldots ,20(items);j=1,\ldots ,n(persons) \end{array}$$ (2)

These are the parameters estimated or referenced in Table 3.

## Supporting Information

S1 Data An excel sheet containing the data. (XLSX)

S1 File Further details about the analysis and results. (PDF)

S1 Video An overview of the entire experiment. (MP4)
