# Supplementary material for: First-Person Perspective Virtual Body Posture Influences Stress: A Virtual Reality Body Ownership Study
Source: PLoS One. 2016 Feb 1;11(2):e0148060. doi: 10.1371/journal.pone.0148060 (PMC4734707; doi:10.1371/journal.pone.0148060)
Supplement: S1 File — (PDF) [file pone.0148060.s002.pdf]

First-person Perspective Virtual Body Posture Influences Stress: A virtual reality body ownership study

Ilias Bergström, Konstantina Kilteni, Mel Slater

S1 File

A. Questionnaires

Table A - Post Experiment Questionnaire with Median and Interquartile Range responses. All responses were on a 1-7 Likert Scale with 1 meaning completely disagree and 7 completely agree.

**(Variable name) Question**	**Comfort Median (IQR)**	**Discomfort Median (IQR)**
(MeDown) Although the virtual body that I saw did not look like me, I felt as if the body I saw when looking down might be my body.	4 (3)	2 (3)
(MeMirror) Although the virtual body that I saw did not look like me, I felt as if the body I saw when looking in the mirror might be my body.	3.5 (3)	2 (2)
(MirrorLegs) Although the virtual body that I saw did not look like me, I felt as if the virtual legs I saw when looking in the mirror might be mine.	2.5 (3)	2 (1)
(MirrorArms) Although the virtual body that I saw did not look like me, I felt as if the virtual arms I saw when looking in the mirror might be mine.	4.5 (3.5)	2 (1)
(LikeMe) I felt as if my virtual body looked like my real body, in terms of skin tone, size, shape and other visual characteristics.	3 (2.5)	3 (3)
(TwoBodiesDown) When I looked down, I had the sensation of having two bodies.	3.5 (3)	3 (3)
(TwoBodiesMirror) When I looked in the mirror, I had the sensation of having two bodies.	2 (2)	3 (1)
(MyPosture) There were moments in which I felt as if my body was in the same posture as that of the virtual body.	2.5 (3)	2 (3)
(Tapping) I felt as if the touches I felt were caused by the yellow balls that I saw were touching my virtual body.	6 (1.5)	4 (4)
(Another) In general I felt that the body belonged to someone else.	4 (3.5)	4 (2)
(Presence) I had the sensation of being inside the room.	6.5 (1)	6 (1)

(Comfort3PP) From the perspective of an external observer, what would you say the level of comfort experienced by the virtual avatar would be?	7 (1)	2 (5)
(Discomfort3PP) From the perspective of an external observer, what would you say the level of discomfort experienced by the virtual avatar would be?	1.5 (1)	5 (4)

It should be noted that most responses were at or below the middle level of the Likert scale. However, in the Comfort condition there was a high level of agreement that the touches felt were caused by the virtual balls touching the virtual body (Tapping). Also there was a very high level of presence in the virtual environment irrespective of condition. Finally the postures were correctly perceived as being comfortable or uncomfortable when considered from a third person perspective (Comfort3PP, Discomfort3PP).

B. Statistical Model

B.1 The Overall Model

The (Bayesian) statistical model is one overall model, where all equations are treated simultaneously rather than as a series of separate models. In other words the Bayesian method returns the joint posterior distribution of all the model parameters. In the following X_i refers to the Condition for the *i*th individual where $X_i = 0$ (Comfort) or 1 (Discomfort). O_i refers to the body ownership score of the *i*th individual. This refers to the variables MeDown or MeMirror (which will be clear from context), and $\tilde{O}$ refers to Another. The overall model has the following components:

- For the **questionnaires scores on body ownership** (MeDown, MeMirror, Another) we use a logistic model (Lunn et al., 2012) (p132-134). The parameters of the linear model that relates the mean of the logistic distribution to the linear model are specified as follows:
    - o $\mu_{Oi} = \beta_{Oo} + \beta_{O1}X_i, i = 1,...,n$ with prior distribution (β_{O0}, β_{O1}): bivariate normal with mean (0,0) and variance-covariance matrix with each variance 1600 and each covariance 160.
- For the **latent comfort score** a normal distribution was used, i.e.

- o $C_i \sim N(\mu_{Ci}, \sigma_C^2)$, where $\mu_{Ci} = CB_i + \beta_{C0} + \beta_{C1}X_i$, CB refers to the baseline comfort, and (β_{C0}, β_{C1}): bivariate normal with mean (0,120), so that the prior probability $P(\beta_{C1} < 0) = 0.0013$ (the probability of a standard normal variate being < -3).

- For **APQ** a log-normal distribution was used, i.e.,
  - o $\log(APQ_i)$: $N(\mu_{Ai}, \sigma_A^2)$,where $\mu_{Ai} = \log(AB_i) + \beta_{A0} + \beta_{A1}X_i$ and (β_{A0}, β_{A1}): bivariate normal with mean vector (0,-120) and variance-covariance matrix as above. *AB* refers to the prior APQ. The -120 was used as the prior mean for β_{A1} since then the prior probability $P(\beta_{A1} > 0) =$ 0.0013 (the probability of a standard normal variate being > 3). The reason for the choice of log-normal is given below.
- For **Heart Rate** a log-normal model was used $\log(H_i)$: $N(\mu_{Hi}, \sigma_H^2)$ where $\mu_{Hi} = \log(HB_i) + \beta_{H0} + \beta_{H1}X_i + \beta_{H2}O_i + \beta_{H3}X_iO_i$ and *HB* represents the heart rate in the baseline, and *O* the body ownership questionnaire response. The prior distribution of the parameters is given by $(\beta_{H0}, \beta_{H1}, \beta_{H2}, \beta_{H3})$: multivariate normal with mean vector (0,0,0,-120) and variance-covariance matrix with all the variances 1600 and all the covariances 160. The value of -120 was chosen for the same reason as above.
- For **NN50** a log-normal distribution for the NN50 values +1 (to avoid log of 0), $\log(N_i + 1)$: $N(\mu_{Ni}, \sigma_N^2)$, with $\mu_{Ni} = \log(NB_i + 1) + \beta_{N0} + \beta_{N1}X_i + \beta_{N2}O_i + \beta_{N3}X_iO_i$ where *NB* is the NN50 in the baseline, and the prior distribution of the parameters in multivariate normal with mean vector (0,0,0,120). The 120 was chosen in order to make the prior probability $P(\beta_{N3} < 0) = 0.0013$.
- For Count a log-normal distribution T_i: $N(\mu_{Ti}, \sigma_T^2)$ was used with $\mu_{Ti} = \log(TB_i) + \beta_{T0} + \beta_{T1}X_i + \beta_{T2}O_i + \beta_{T3}X_iO_i$, where *TB* is the baseline count, and the joint distribution of the parameters is multivariate normal with mean vector (0,0,0,120) and variance-covariance matrix as above.

The distribution of the variances $\sigma_C^2, \sigma_A^2, \sigma_H^2, \sigma_N^2, \sigma_T^2$ were given as independent Gamma distributions with parameters (0.001, 0.001) in the JAGS / BUGS specification.

It should be noted that as well as the given values of the variances (1600) specified above, a range of other values were used (ranging up to 10,000) with no difference in results. Also the mean vector for the prior distributions were chosen to be heavily biased against our hypotheses, with probabilities of only 0.0013 as explained above.

Under this method readers are free to interpret the probabilities of the hypotheses in different ways of course. We have used the following: We start with a strong bias against each of the hypotheses - the prior probability assigned is about 1/1000. If the posterior probabilities are around the 50% range then we would say that from being biased against the hypothesis we move to a 50-50 probability and more evidence is needed. Probabilities above 70% we refer to as 'little' or 'some' evidence in favor of the hypothesis. Above 80% we use the term 'good evidence'. Above 90% 'strong evidence', and in one case with the probability almost 1 we use the term 'overwhelming evidence'.

B.2 Choice of Log-normal distributions

The latent comfort variables LComfort, LComfortBase are designed to be normally distributed from the IRT method. For the remaining variables it was found that normal distributions for the likelihoods did not take account of some extreme outliers. Looking at the baseline distributions of the response variables it was clear that log-normal was more appropriate than normal, and there is less problem with outliers. The DIC for the model is 329.

C. Posterior distributions of the model parameters

C.1 Coefficients

The following Figures should be examined in relation to Table 1, they give the posterior distributions of the model parameters.

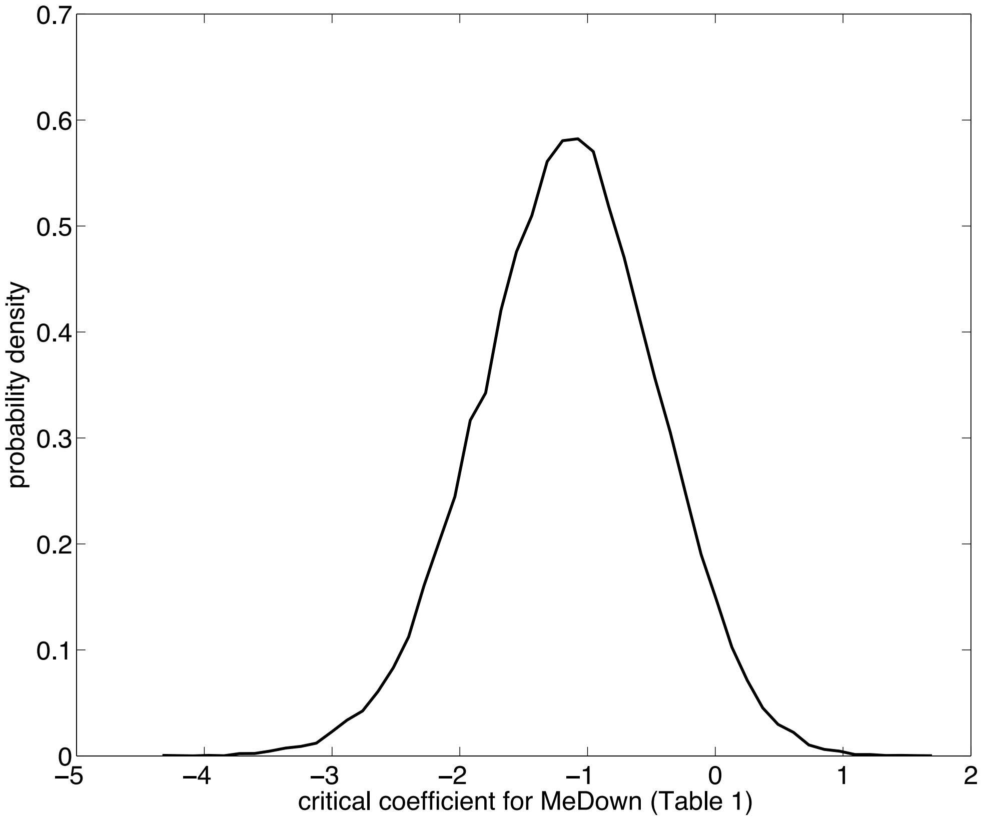

Figure A - Posterior distribution of β_{O1} the coefficient of the factor Condition (Comfort=0, Discomfort = 1) in the model for MeDown in Table 1 .

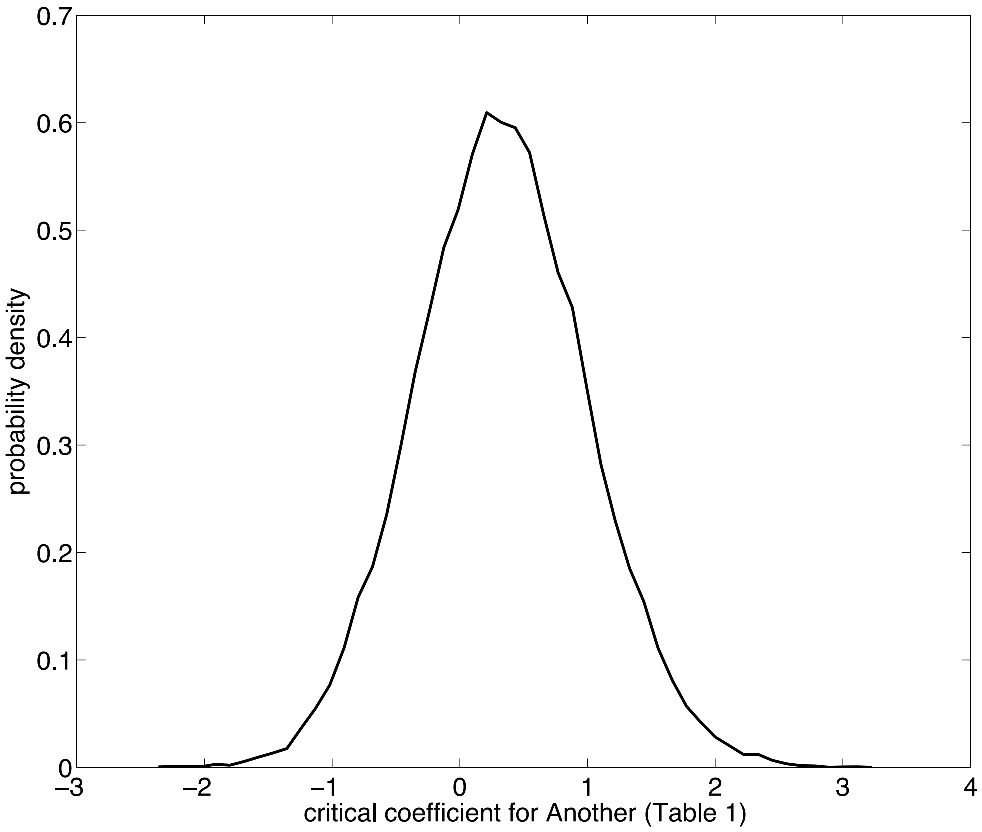

Figure B - Posterior distribution of β_{NO1} the coefficient of the factor Condition (Comfort = 0, Discomfort = 1) in the model for Another in Table 1.

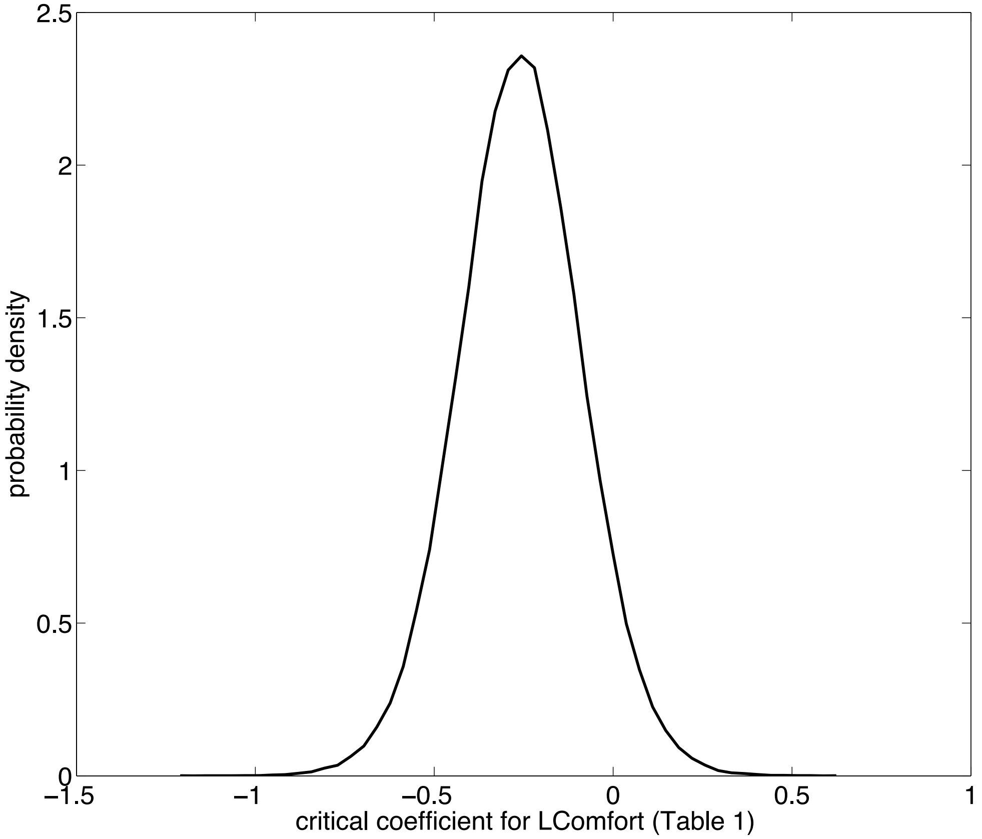

Figure C - Posterior distribution of β_{C1} the coefficient of the factor Condition (Comfort = 0, Discomfort = 1) in the model for LComfort in Table 1..

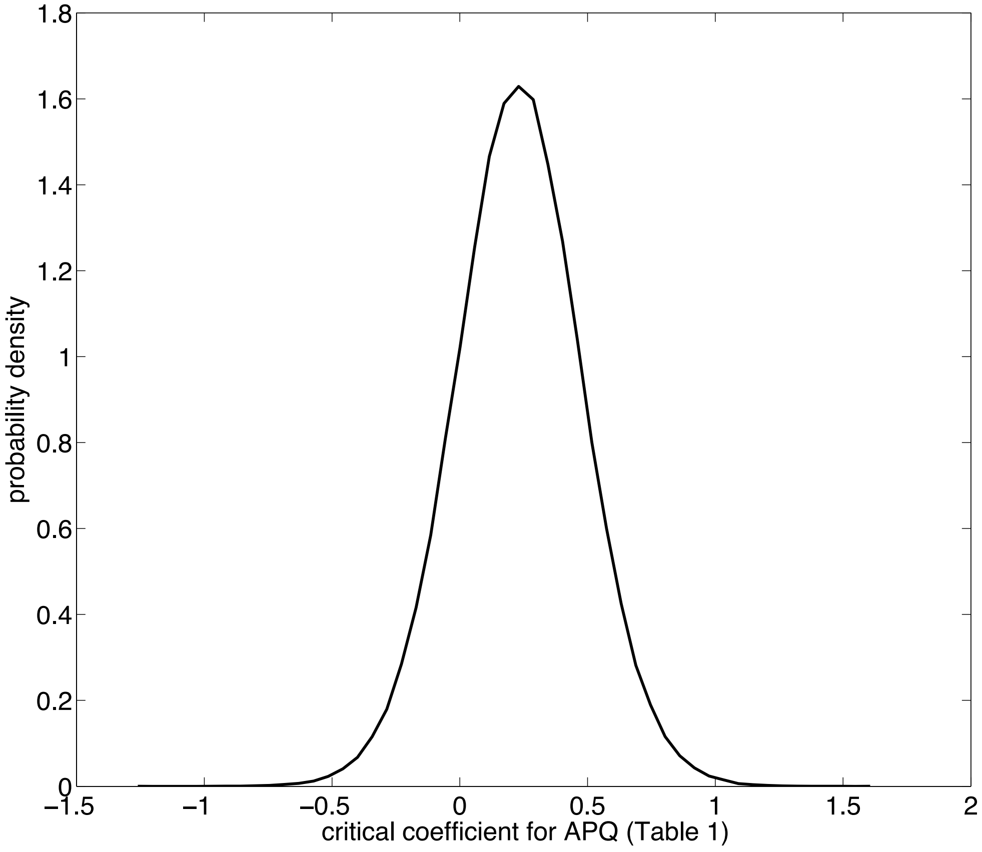

Figure D - posterior distribution of β_{A1} the coefficient of the factor Condition (Comfort = 0, Discomfort = 1) in the model for APQ in Table 1.

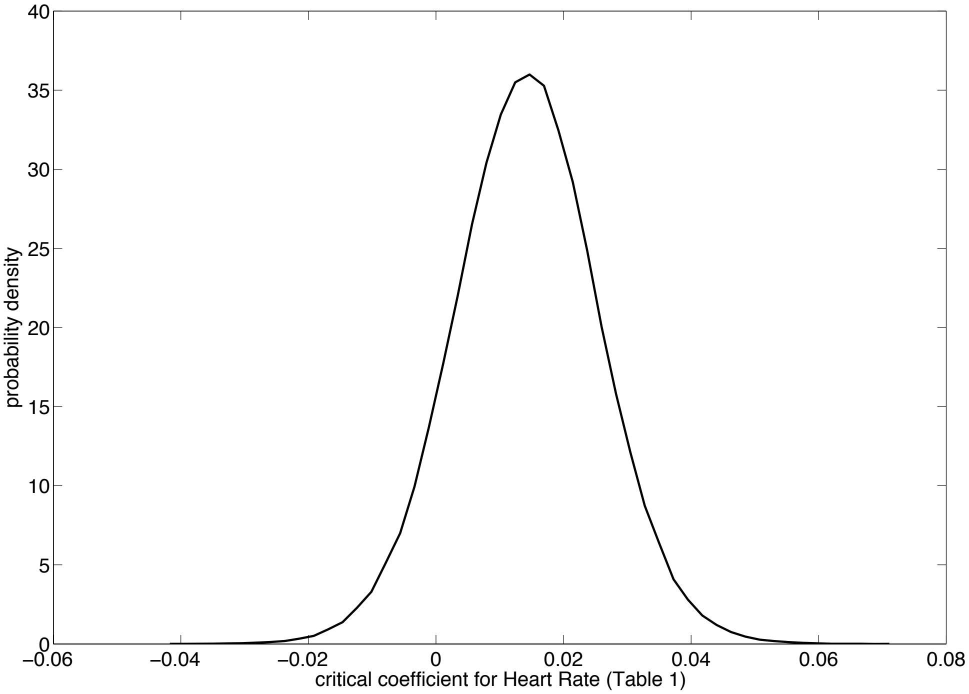

Figure E - posterior distribution of β_{H3} the coefficient of the factor Condition (Comfort = 0, Discomfort = 1) in the model for Heart Rate in Table 1.

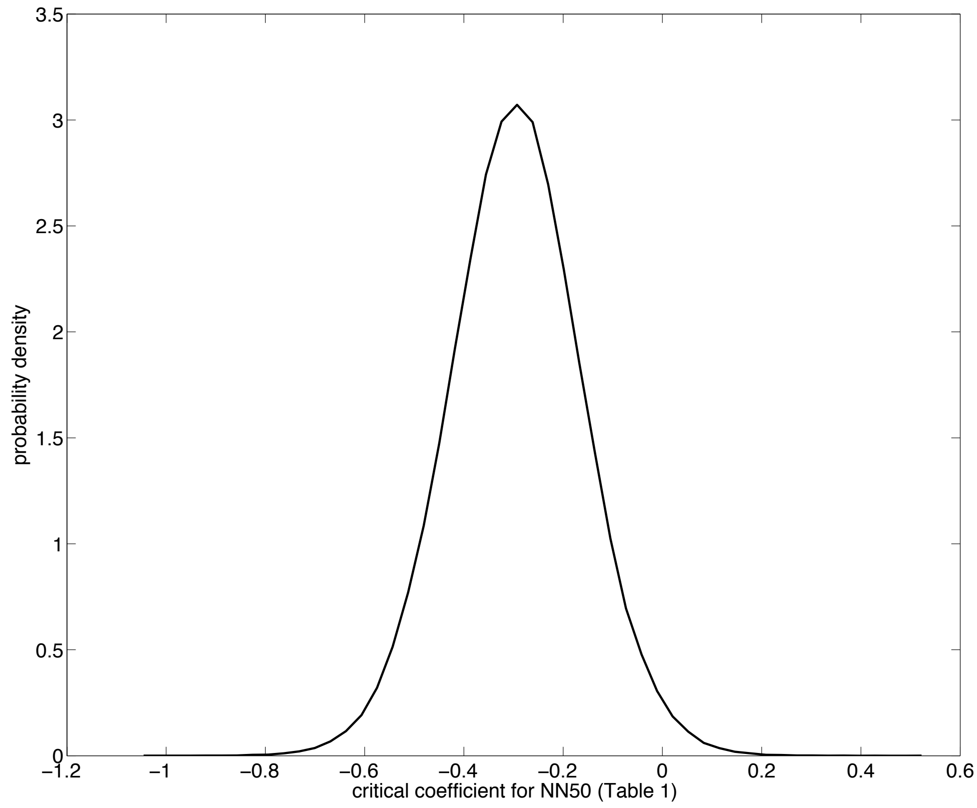

Figure F - posterior distribution of β_{N3} the coefficient of the factor Condition (Comfort = 0, Discomfort = 1) in the model for NN50 in Table 1.

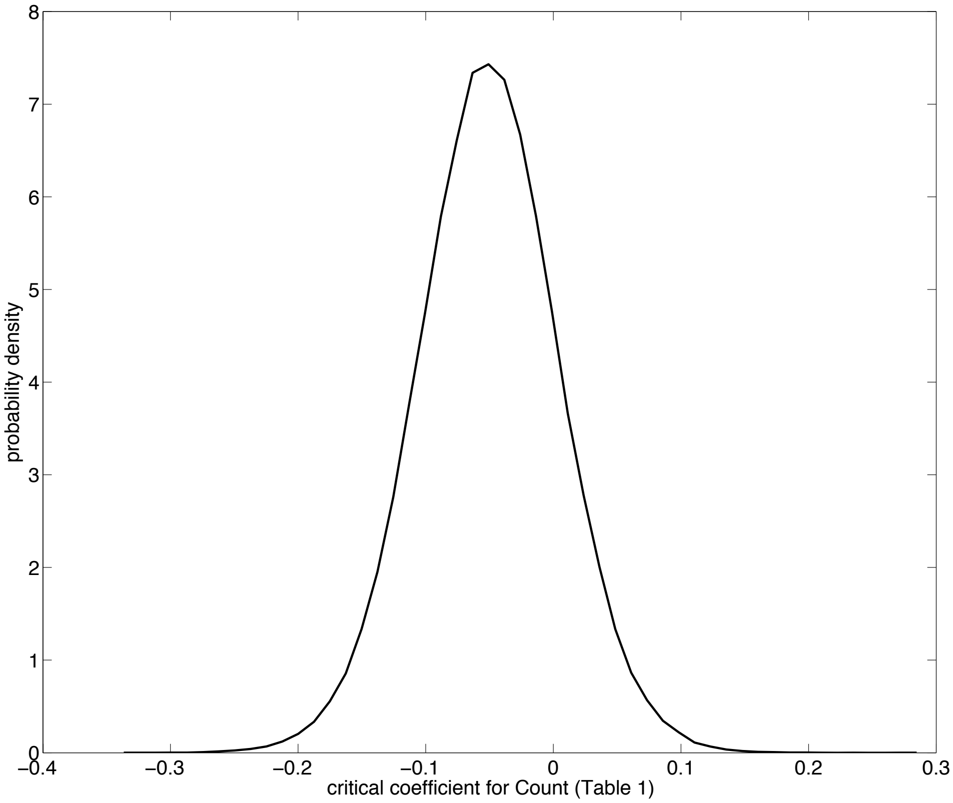

Figure G - posterior distribution of β_{T3} the coefficient of the factor Condition (Comfort = 0, Discomfort = 1) in the model for Count in Table 1.

C.2 Standard Deviations

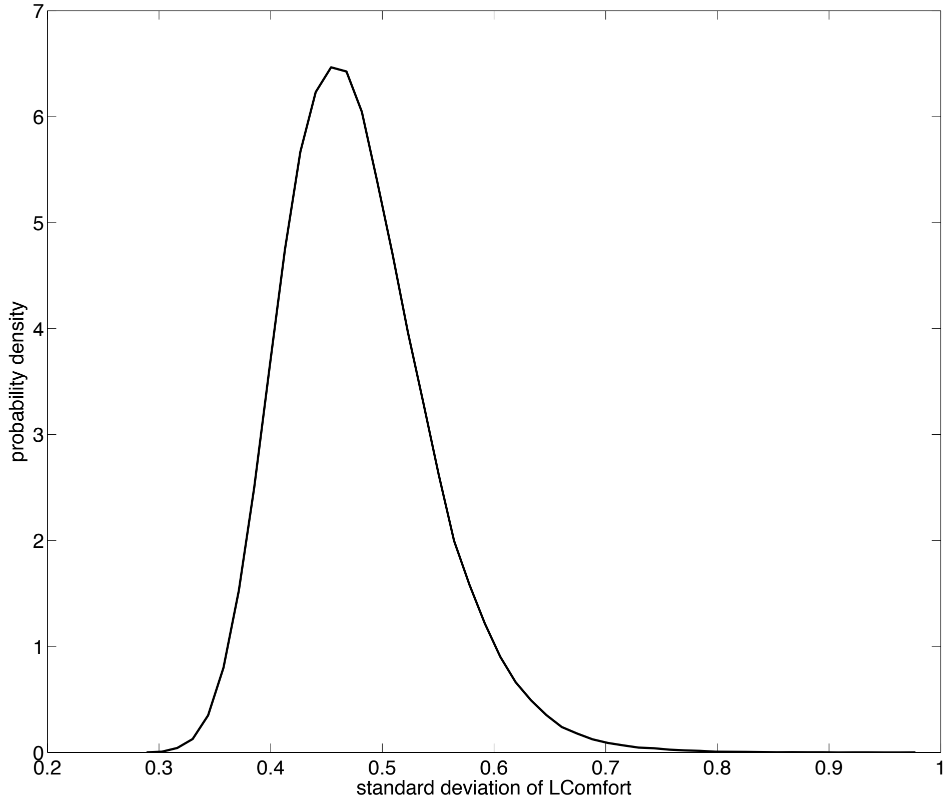

Figure H - Posterior distribution of the standard deviation of LComfort (σ_C) in the model for LComfort in Table 1

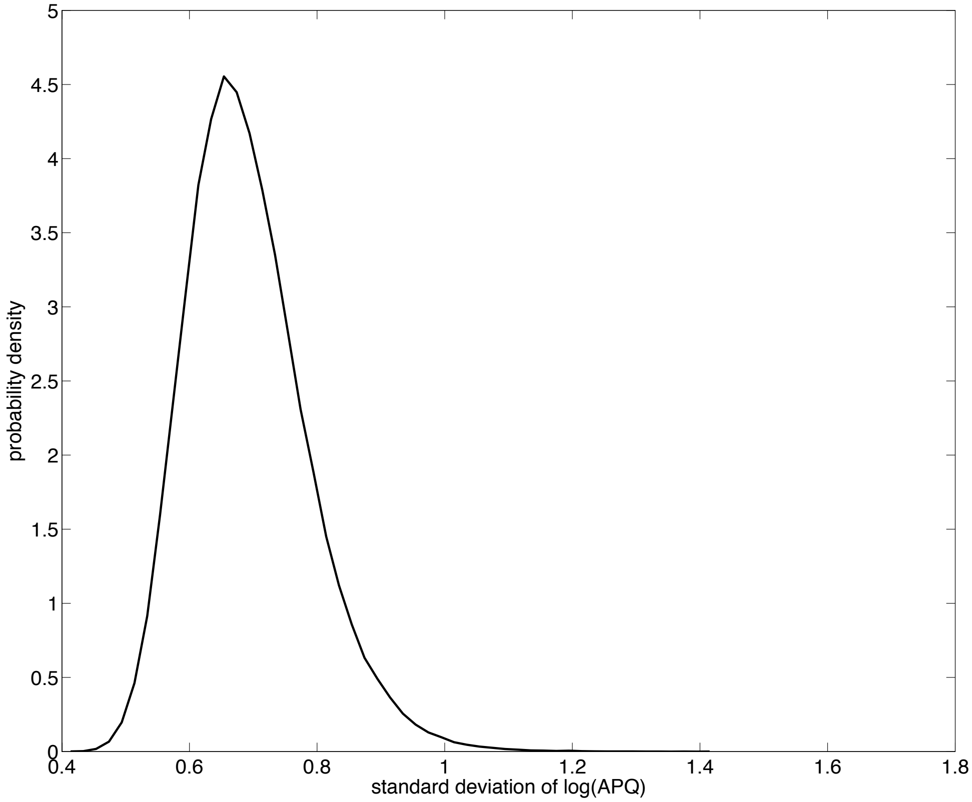

Figure I - Posterior distribution of the standard deviation of log(APQ) (σ_A) in the model for APQ in Table 1

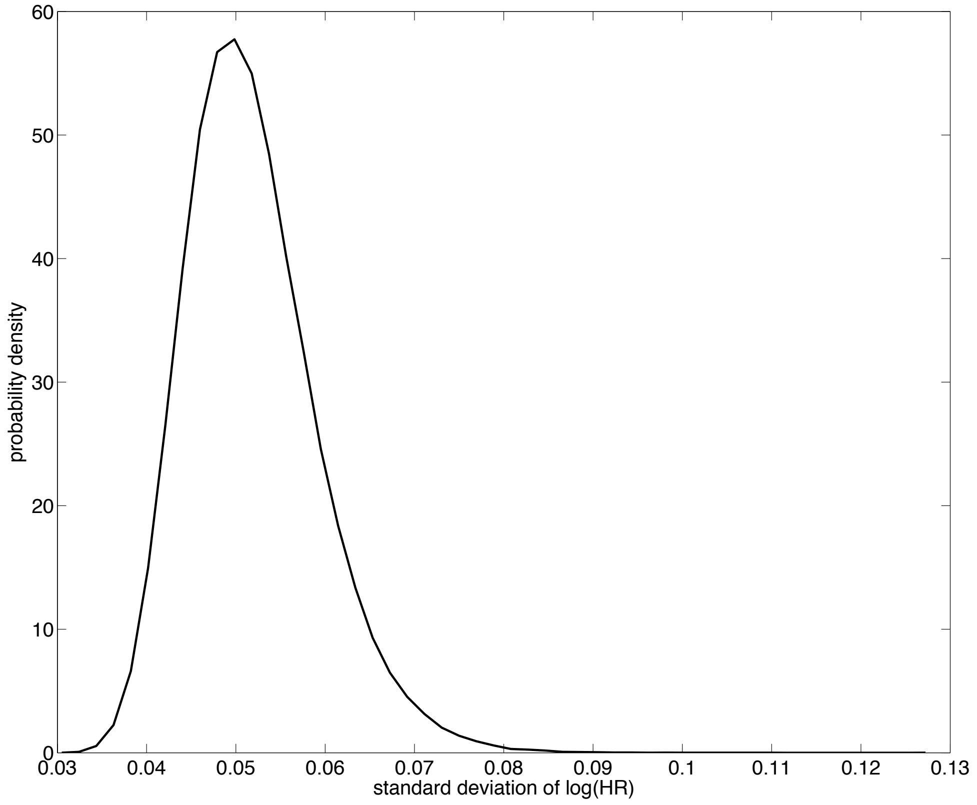

Figure J - Posterior distribution of the standard deviation of log(Heart Rate) (σ_{HR}) in the model for Heart Rate in Table 1

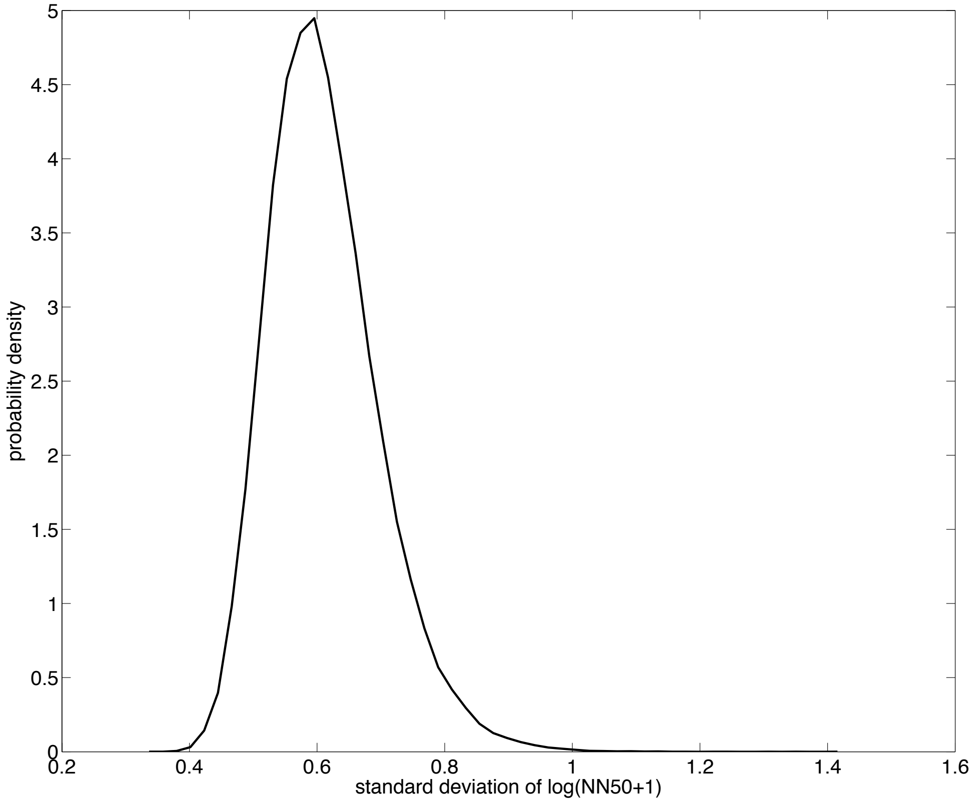

Figure K - Posterior distribution of the standard deviation of log(NN50+1) (σ_N) in the model for NN50 in Table 1

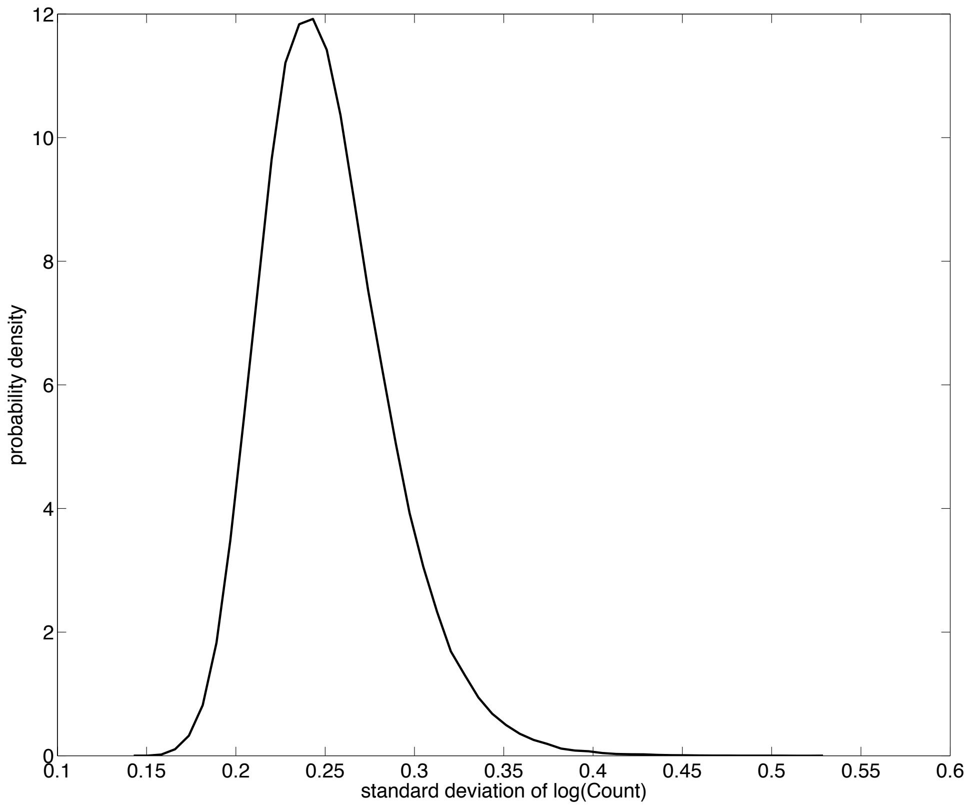

Figure L - Posterior distribution of the standard deviation of log(Count) (σ_T) in the model for Count in Table 1

D. Convergence

Each Markov Chain Monte Carlo simulation was run 7 times (according to convention) with a sample size of 30,000 observations and a burn-in of 3000. All Rhat values - measuring consistency between the results of the 7 chains - were equal to 1 meaning that convergence was obtained.

E. Fit of the Model

To examine the goodness of fit we compare predicted with observed values. For each of the quantitative variables we can obtain the posterior predicted distribution of the variable conditional on the observed data for each individual participant. For a predicted value for any particular

individual we take the mean of that distribution. Below, each of the quantitative variables are considered in turn, and it can be seen that the fits are good.

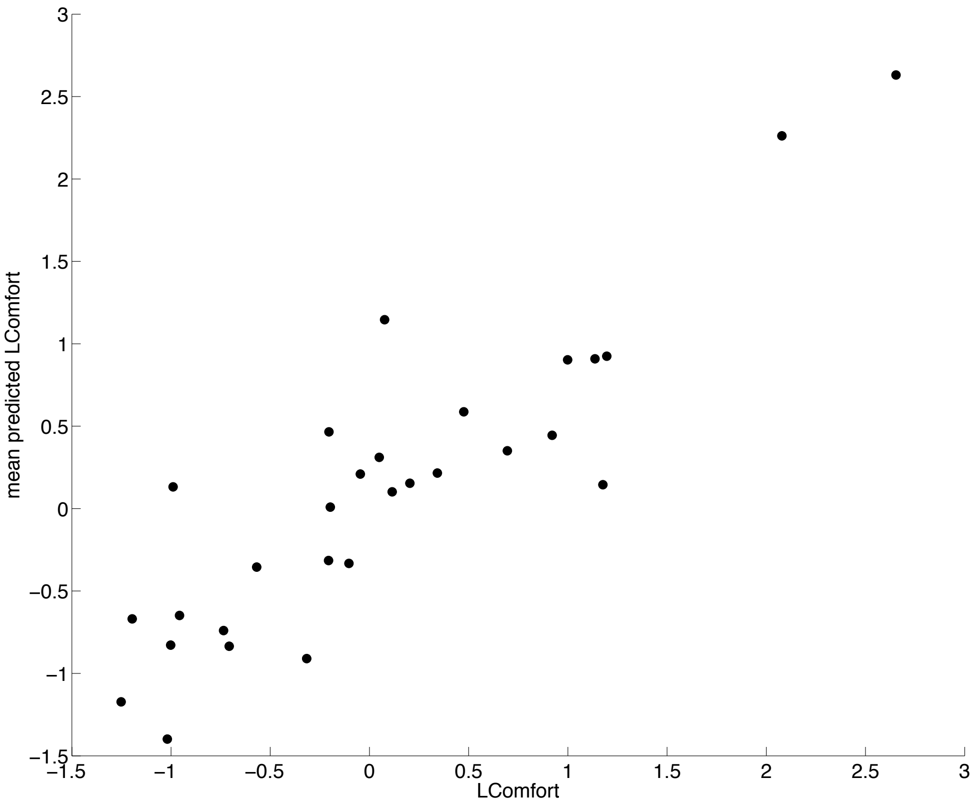

Figure M - mean predicted values of LComfort by observed values, r = 0.90, n = 31

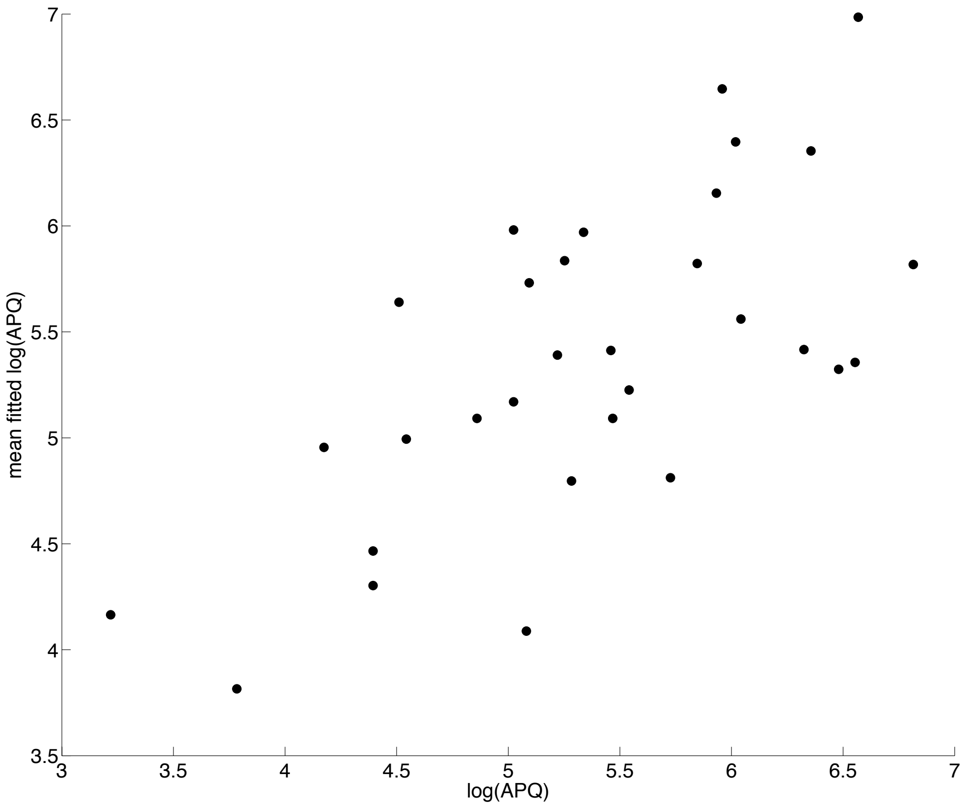

Figure N - mean predicted values of log(APQ) by observed values, r = 0.68, n = 31

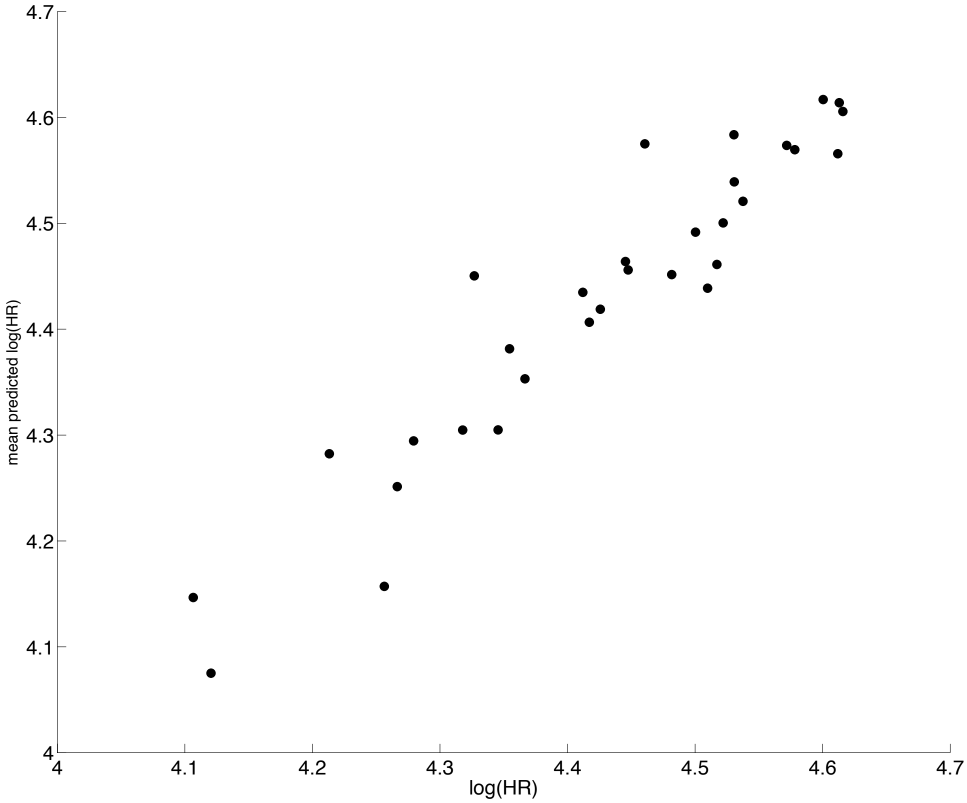

Figure O - mean predicted values of log(HR) by observed values, r = 0.95, n = 31

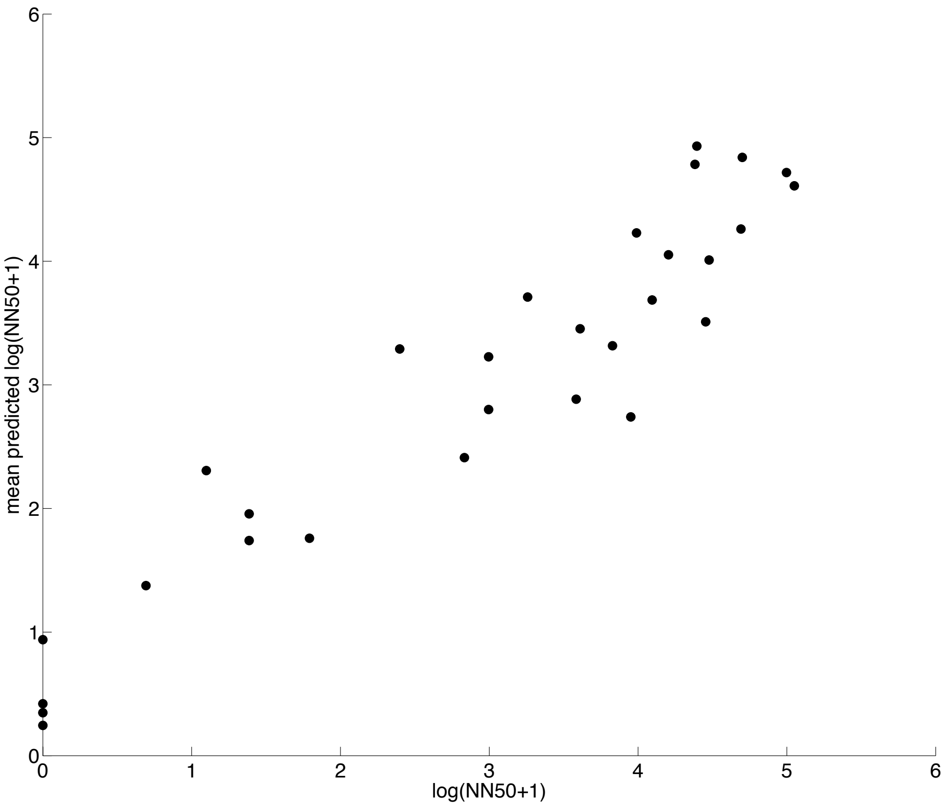

Figure P - mean predicted values of log(NN50+1) by observed values, r = 0.95, n = 31

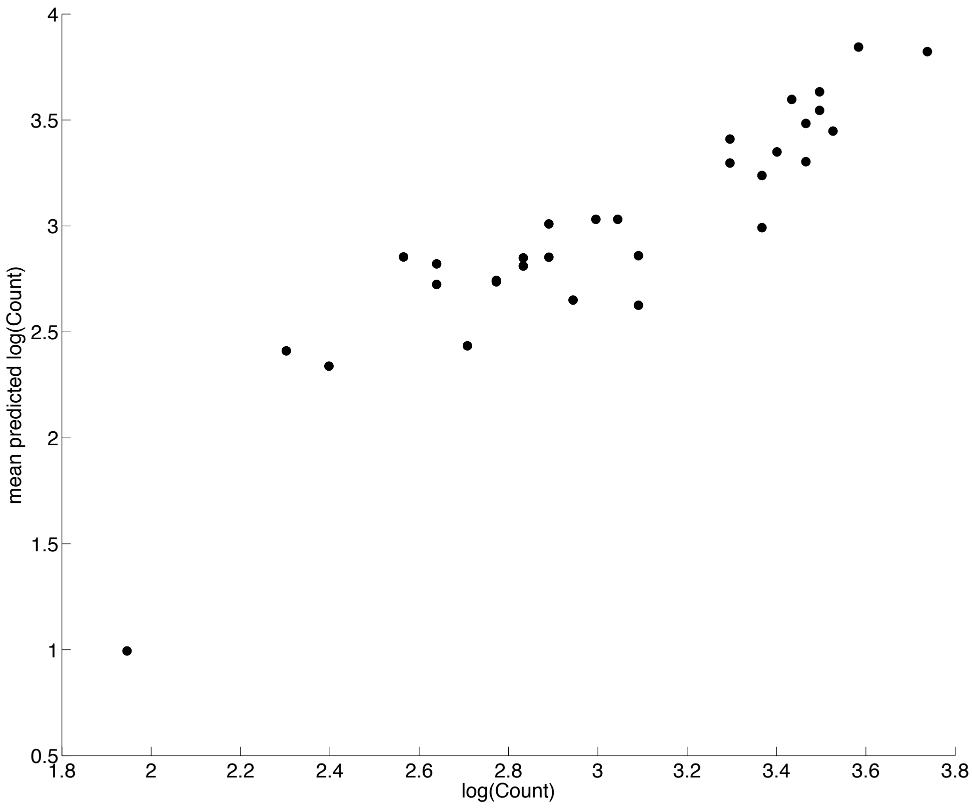

Figure Q - mean predicted values of log(Count) by observed values, r = 0.91, n = 31

F. Alternative Models

Here we consider two alternative models - the first replaces MeDown by MeMirror, Comfort by Discomfort, and ComfortBase by DiscomfortBase. Here we would expect similar results to those of Table 1 in the paper. Table S2 shows that overall the results are the same except that the evidence does not suggest that the latent discomfort is greater in the Discomfort condition than in the Comfort condition. Replacing MeDown by MeMirror leads to only one main change which is that the Count variable is likely to have a positive interaction term between MeMirror and Condition rather than negative as would be expected, which is likely due to one outlying observation in MeMirror. The DIC for this model is 349 which is similar to that of the original model.

Table B - Results of the Statistical Analysis on all the responses related to Comfort - replacing Comfort by Discomfort and MeDown by MeMirror in Table 1.

Throughout *B* refers to Baseline, e.g. *D* is the Discomfort variable with respect to the experimental period and *DB* refers to the variable Discomfort in the Baseline. X is the experimental condition (X=0 Comfort, X=1 Discomfort). O refers to MeMirror.

**Response Variable, individual *i***	**Link between mean and linear model**	**Hypotheses is (H) on the parameter of interest**	**P(H|D) D=data. Priors = 0.001**	**Interpretation**
**MeMirror** O_i	$\mu_{Oi} = \beta_{O0} + \beta_{O1}X_i$	$\beta_{O1} < 0$	0.94	Strong evidence that Ownership is less under the Discomfort condition.
**Another** NO_i	$\mu_{NOi} = \beta_{NO0} + \beta_{NO1}X_i$	$\beta_{NO1} > 0$	0.68	Some evidence that Non-ownership is greater in the Discomfort condition.
**Discomfort** D_i	$\mu_{Di} = DB_i + \beta_{D0} + \beta_{D1}X_i$ $\log(D_i) \sim N(\mu_{Di}, \sigma^2_D)$	$\beta_{D1} > 0$	0.44	Little evidence that LDiscomfort is greater in the Discomfort condition relative to baseline
**APQ** A_i	$\mu_{Ai} = \log(AB_i) + \beta_{A0} + \beta_{A1}X_i$ $\log(A_i) \sim N(\mu_{Ai}, \sigma^2_A)$	$\beta_{A1} > 0$	0.83	Good evidence that APQ is greater relative to baseline in the Discomfort condition.
**Heart Rate** H_i	$\mu_{Hi} = HB_i + \beta_{H0} + \beta_{H1}X_i + \beta_{H2}O_i + \beta_{H3}X_i \cdot O_i$	$\beta_{H3} > 0$	0.97	Strong evidence that HR is positively associated with BOI (MeMirror) in the Discomfort condition.
**NN50** N_i	$\log(\mu_{Ni}) = \log(NB_i + 1) + \beta_{N0} + \beta_{N1}X_i + \beta_{N2}O_i + \beta_{N3}X_i \cdot O_i$	$\beta_{N3} < 0$	0.9998	Overwhelming evidence that NN50 is negatively associated with

				BOI (MeMirror) in the Discomfort condition.
**Count** T_i	$\mu_{Ti} = TB_i + \beta_{T0} + \beta_{T1}X_i + \beta_{T2}O_i + \beta_{T3}X_i \cdot O_i$ $\log(T_i) \sim N(\mu_{Ti}, \sigma_T^2)$	$\beta_{T3} < 0$	0.32	Some evidence that the number of correct counts is positively associated with BOI (MeMirror) in the Discomfort condition.

The second model is a 'counter example' where in contrast to Table 1 we replace MeDown by Another. Here we would expect the relationships of HR, NN50 and Count with this ownership variable to be different in comparison with MeDown (Table 1). The results are shown in Table S3. It can be seen that the relationships with HR and Count diminishes to around the 50% level, and with NN50 it reverses. The DIC is 341.

Table C - Results of the Statistical Analysis on all the responses related to Comfort - replacing MeDown by Another, and Another by TwoBodiesDown in relation to Table 1.
Throughout *B* refers to Baseline, e.g. *HR* is the heart rate variable with respect to the experimental period and *HRB* refers to heart rate in the Baseline. X is the experimental condition (X=0 Comfort, X=1 Discomfort). O refers to Another.

**Response Variable, individual *i***	**Link between mean and linear model**	**Hypothesis (H) on the parameter of interest**	**P(H|D) D=data**	**Interpretation**
**Another** NO_i	$\mu_{NOi} = \beta_{NO0} + \beta_{NO1}X_i$	$\beta_{NO1} > 0$	0.67	Some evidence that Non-ownership (Another) is greater under the Discomfort condition.
**TwoBodies Down** $O2_i$	$\mu_{O2i} = \beta_{O20} + \beta_{O21}X_i$	$\beta_{O21} > 0$	0.42	Little evidence that Non-ownership (TwoBodiesDown) is influenced by the Comfort

				conditions.
**Comfort** C_i	$\mu_{Ci} =$ $CB_i + \beta_{C0} + \beta_{C1}X_i$ $\log(C_i) : N(\mu_{Ci}, \sigma_C^2)$	$\beta_{C1} < 0$	0.93	Strong evidence that Comfort is less relative to the baseline in the Discomfort condition.
**APQ** A_i	$\mu_{Ai} =$ $\log(AB_i) + \beta_{A0} + \beta_{A1}X_i$ $\log(A_i) : N(\mu_{Ai}, \sigma_A^2)$	$\beta_{A1} > 0$	0.83	Good evidence that APQ is greater relative to baseline in the Discomfort condition.
**Heart Rate** H_i	$\mu_{Hi} =$ $HB_i + \beta_{H0} + \beta_{H1}X_i +$ $\beta_{H2}\tilde{O}_i + \beta_{H3}X_i \cdot \tilde{O}_i$	$\beta_{H3} > 0$	0.52	Little evidence of any association between HR and the non-ownership in the Discomfort condition.
**NN50** N_i	$\log(\mu_{Ni}) =$ $\log(NB_i + 1) + \beta_{N0} + \beta_{N1}X_i +$ $\beta_{N2}\tilde{O}_i + \beta_{N3}X_i \cdot \tilde{O}_i$	$\beta_{N3} < 0$	0.28	Some evidence that NN50 is positively associated with non-ownership in the Discomfort condition.
**Count** T_i	$\mu_{Ti} =$ $TB_i + \beta_{T0} + \beta_{T1}X_i +$ $\beta_{T2}\tilde{O}_i + \beta_{T3}X_i \cdot \tilde{O}_i$ $\log(T_i) : N(\mu_{Ti}, \sigma_T^2)$	$\beta_{T3} < 0$	0.56	Little evidence that the number of correct counts is by non-ownership in the Discomfort condition.

G. The Structure of Discomfort

Table S4 shows the equivalent of Table 3 for the discomfort scores. While the baseline results on discrimination are quite similar to those of comfort the experiment scores show some marked differences, and differences from the baseline discomfort scores. For example, shoulders, upper back and nape of the neck have high discrimination values in the experimental condition, but quite low values in the baseline.

Table D - Estimates and their Standard Errors of the Discrimination Parameter for each of the 20 Items in the Body Map for the Comfort Questions. D(s≥4) is the rank order of the difficulty parameter for obtaining a score of at least 4 from the IRT model, where 1 is the most and 20 the least difficult (backs of legs did not have a score of 4 and hence the maximum is 19).

Baseline				Experiment			
	Coef	S.E.	D(s≥4)		Coef	S.E.	D(s≥4)
Backs of legs	6.15	2.26	9	Shoulders	6.07	1.96	13
Legs	5.84	2.17	12	Backs of shoulders	5.23	1.58	16
Arms	5.07	1.67	5	Upper back	4.37	1.21	19
Backs of arms	3.28	1.02	6	Nape of neck	3.44	0.93	17
Thighs	2.81	0.90	2	Chest	3.43	1.04	12
Chest	2.63	0.80	14	Lower back	2.96	0.82	14
Abdomen	2.62	0.82	11	Neck	2.94	0.83	15
Backs of thighs	2.62	0.85	8	Backs of arms	2.50	0.78	11
Feet	1.93	0.71	10	Arms	2.39	0.75	8
Shoulders	1.72	0.56	13	Abdomen	2.29	0.72	10
Backs of shoulders	1.71	0.56	18	Head	2.08	0.61	18
Backs of feet	1.70	0.65	4	Backs of thighs	2.00	0.65	9
Buttocks	1.52	0.58	3	Backs of legs	1.88	0.64	-
Head	1.43	0.50	16	Thighs	1.85	0.62	6
Hands	1.37	0.52	7	Buttocks	1.61	0.56	7
Lower back	1.36	0.49	19	Legs	1.41	0.55	4
Backs of hands	1.27	0.48	1	Backs of hands	0.75	0.39	2
Nape of neck	0.98	0.41	17	Feet	0.73	0.40	5
Neck	0.97	0.42	15	Hands	0.67	0.39	1
Upper back	0.93	0.41	20	Backs of feet	0.62	0.39	3

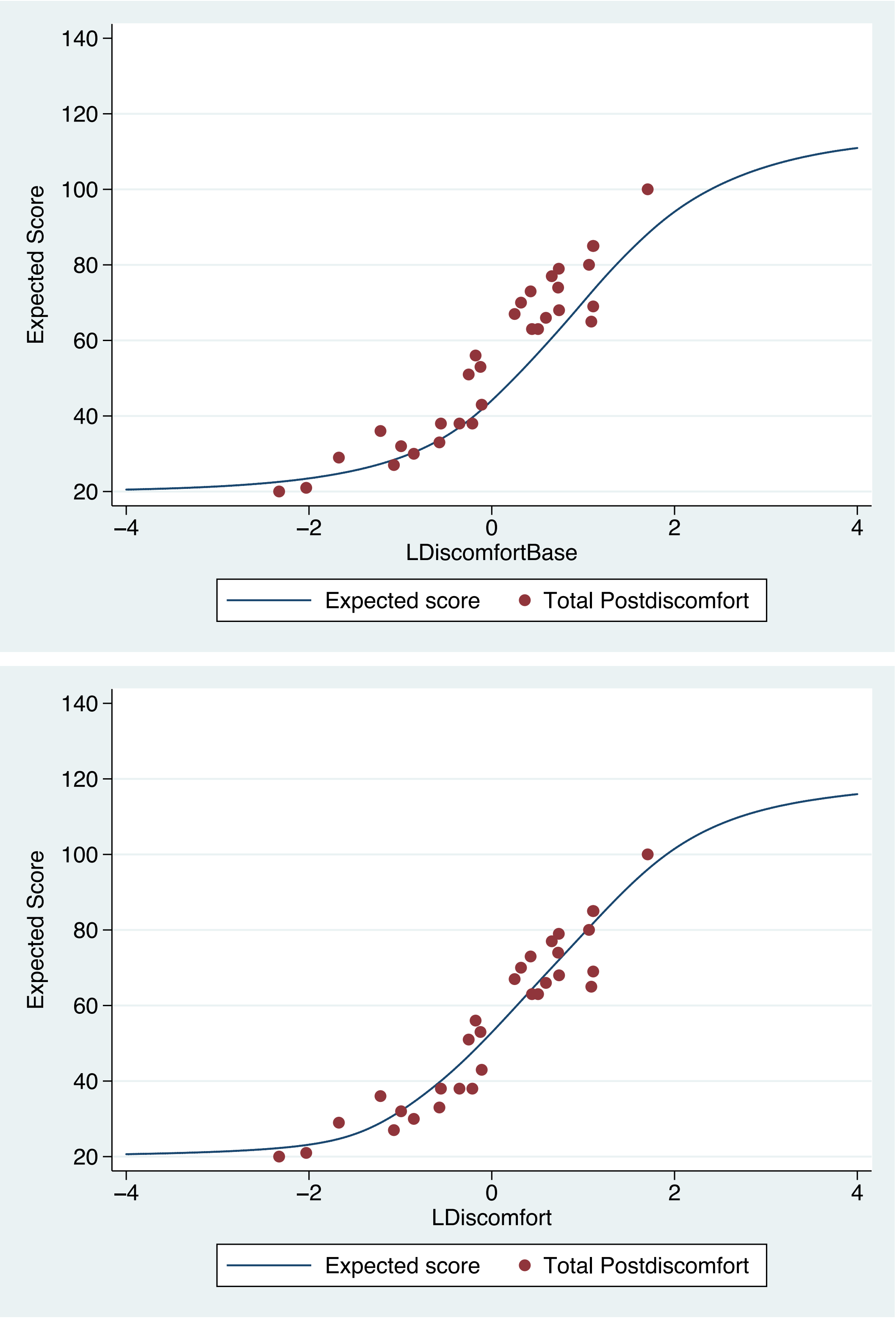

Figure R - Expected discomfort scores from the IRT model for the Discomfort condition

Supporting Reference

LUNN, D., JACKSON, C., BEST, N., THOMAS, A. & SPIEGELHALTER, D. 2012. *The BUGS book: A practical introduction to Bayesian analysis*, CRC press.
